# A Feasibility Assessment of the FDA Adverse Event Reporting System for the Detection of Cannabis‐Related Safety Signals

**DOI:** 10.1002/pds.70392

**Published:** 2026-05-13

**Authors:** Priscilla O. M. V. Lopes, Cory S. Harris, Christopher A. Gravel

**Affiliations:** ^1^ School of Epidemiology and Public Health University of Ottawa Ottawa Ontario Canada; ^2^ Department of Biology University of Ottawa Ottawa Ontario Canada; ^3^ Department of Chemistry and Biomolecular Sciences University of Ottawa Ottawa Ontario Canada; ^4^ Department of Mathematics and Statistics University of Ottawa Ottawa Ontario Canada; ^5^ Data Literacy Research Institute University of Ottawa Ottawa Ontario Canada

**Keywords:** adverse events, cannabinoids, cannabis, disproportionality analysis, FAERS, pharmacovigilance

## Abstract

**Background:**

The applicability of spontaneous reporting systems such as the US Food and Drug Administration Adverse Event Reporting System (FAERS) to detect cannabis‐related safety signals remains unclear due to the potential for discrepant reporting patterns between pharmaceutical and non‐pharmaceutical cannabis‐derived products (CDPs).

**Methods:**

We conducted a descriptive analysis of seven groups of CDP reports submitted to FAERS between 1999 and 2023 to investigate product definitions and reporting patterns. We then performed hypothesis‐free disproportionality analyses using reporting odds ratio, proportional reporting ratio, and information component for pharmaceutical cannabidiol (CBD) and non‐pharmaceutical CBD reports to assess differences in signal detection profiles, potential exposure misclassification, and the influence of reporting context.

**Results:**

We identified 42 530 reports related to CDPs, characterized by highly heterogeneous terminology and variable reporting patterns by product type, reflecting the real‐world CDP usage. Epidiolex reports often involved pediatric patients, whereas non‐pharmaceutical CBD reports were more frequently associated with older adults and concomitant product use. Disproportionality analysis showed divergent signal profiles, with strong seizure‐related events predominating for Epidiolex and a broader range of signals, including neoplasm‐related and neurological events, observed for non‐pharmaceutical CBD. These differences likely reflected variations in CDP indication and utilization and reporting behaviors.

**Conclusions:**

This study showed that signal detection using FAERS has potential feasibility for CDP safety surveillance. However, unique challenges related to exposure definitions, reporting patterns, motivation for utilization, and the need for a robust study design must be addressed to ensure reliable safety signal detection.

## Key Points

- Self‐reported cannabis‐derived product (CDP) terminology and exposure definitions were highly heterogeneous in FAERS, complicating product identification and classification.
- Reporting patterns and motivations for utilization differed by CDP type, including patient populations and concomitant product use.
- Pharmaceutical and non‐pharmaceutical cannabidiol exhibited distinct signal detection profiles, with seizure‐predominant signals for pharmaceutical CBD versus broader neoplasm/neurologic signals for non‐pharmaceutical CBD.
- Collectively, heterogeneity, utilization context, and profile differences introduced challenges for FAERS‐based CDP surveillance beyond standard disproportionality limitations.
- These findings emphasized the need for rigorous methodological design and systematic exposure classification to improve the reliability of CDP safety surveillance.

## Executive Summary

Self‐reports of adverse health events suspected to be related to medications are collected in the US FDA Adverse Event Reporting System (FAERS) and screened for early signals of potential side effects. The feasibility of using FAERS to monitor potential adverse drug reactions to cannabis‐derived products (CDPs) is unclear, as individual cannabinoids are utilized in different ways for varying reasons, described with different names, and include both prescription and non‐prescription forms, unlike other medications in the database. Reports concerning seven groups of CDPs submitted to FAERS from 1999 to 2023 were summarized to explore product descriptions and reporting patterns. Potential safety signals were then screened for prescription cannabidiol and non‐prescription cannabidiol using standard methods. Significant variation in product description was observed, and reporting patterns varied by product type. Signal detection profiles also differed between prescribed and non‐prescribed cannabidiol. These differences may reflect patterns of use, indications, and reporting context. We conclude that while FAERS can contribute to CDP safety surveillance, reliable interpretation requires clearer product definitions, methods of classification, and study designs that account for the unique reporting patterns, which pose challenges beyond the usual limitations of drug safety surveillance in this database.

## 1 Introduction

Legal and regulatory changes over the past two decades have altered the accessibility, composition, and usage of cannabis and cannabis‐derived products (CDPs) globally. In addition to pharmaceutical‐grade CDPs such as Epidiolex (cannabidiol [CBD]) [1], dronabinol (Marinol, Syndros) [2, 3], nabilone (Cesamet) [4], and nabiximols (Sativex) [5], a range of non‐pharmaceutical CDPs is available through regulated and unregulated markets. This expansion in product types, formulations, and user populations has raised new challenges for pharmacovigilance, particularly in identifying potential adverse drug reactions (ADRs) associated with diverse CDP exposures [6, 7].

Despite the increasing use of both pharmaceutical and non‐pharmaceutical CDPs, approaches for post‐market safety surveillance remain poorly characterized. Clinical trials for pharmaceutical CDPs are typically restricted to standardized formulations, narrow indications, and specific patient populations limiting their generalizability [8]. For non‐pharmaceutical CDPs, the absence of standardized dosing and manufacturing controls complicates safety assessment, as utilization data needed to contextualize adverse event (AE) reports cannot be reliably captured through conventional pharmacoepidemiological data sources [9]. Therefore, spontaneous reporting systems, like the US FDA Adverse Event Reporting System (FAERS), may support the detection of previously unknown CDP safety signals [10].

The multifaceted nature of CDP utilization complicates safety surveillance in FAERS using algorithms designed for medications without a recreational/non‐pharmaceutical counterpart. Challenges include inconsistent terminology within product type, coexistence of therapeutic and recreational use, and highly variable formulations and product compositions, particularly for non‐pharmaceutical CDPs. Varying regulatory oversight exacerbates these challenges, as legal restrictions may discourage AE reporting. Confounding by association may be present due to polysubstance use [11]. Together, the heterogeneity and complexity of the CDP landscape may influence reporter behavior and population‐level reporting patterns, with specific forms of reporting bias that depend on CDP type, whether pharmaceutical or recreational/non‐pharmaceutical [11].

Recently, the READUS‐PV guideline presented standards for disproportionality analyses in spontaneous reports for bias reduction and transparency [12]. To date, no study has systematically examined whether FAERS can support reliable signal detection for cannabis‐related AEs, and whether consensus guideline recommendations directly apply to CDPs or need adaptation. Understanding methodological constraints for CDP signal detection, and whether it carries a different potential for bias than other therapeutic products, is critical for optimizing cannabis science and surveillance efforts.

We assessed the feasibility of using FAERS for cannabis safety surveillance by first conducting a descriptive analysis of CDP‐related reports between 1999 and 2023 to explore the diversity of CDP terminology and reporting patterns. We then conducted hypothesis‐free signal detection of CBD‐containing products, reported as pharmaceutical or non‐pharmaceutical, to assess whether disproportionality reflects the cannabinoid itself or context‐dependent reporting introduced by ambiguous CDP definitions.

## 2 Methods

### 2.1 Data Source

We used the FAERS database from the first quarter (Q1) of 1999 to Q1 2023. Submission of suspected ADR reports to the FDA is voluntary for healthcare professionals, patients, and consumers, but is mandatory for product manufacturers. These reports include demographics, drug information (including the suspected role in the AE), clinical information about the reaction, outcomes, report sources, and indications and are publicly available in seven files linked through a unique identifier [13]. Suspected reactions are coded using Medical Dictionary for Regulatory Activities (MedDRA) terminology at the preferred term (PT) level, which links to a system organ class (SOC; hierarchical structure by etiology, site and purpose) [14].

We linked the quarterly data by case identification number, keeping the information in the most recent update linked to the initial report date. Reports were included regardless of the suspected role the reporter assigned to the CDP.

### 2.2 Exposure Definition

FAERS does not apply a standardized coding system specific to cannabinoid‐containing products. Therefore, drug names are recorded as reported. In 2025, the FDA revised MedWatch forms to include ‘Cannabinoid Hemp Products’ as an explicit product type category, though this classification was not available during our study period [15]. Due to the lack of standardized nomenclature for CDPs in FAERS and to improve the specificity of exposures, we searched for character strings associated with pharmaceutical and non‐pharmaceutical formulations of cannabinoids. Given that varying terminologies were used, we included medical and non‐medical names of major cannabinoids. The following terms were searched: ‘cannab’, ‘canab’, ‘mariju’, ‘marih’, ‘thc’, ‘cbd’, ‘nabixi’, ‘dronab’, ‘nabilo’, ‘cesam’, ‘syndros’, ‘marino’, ‘epidiol’, and ‘sative’. The string ‘cannab’ was selected to broadly capture cannabinoid‐related terms, including full chemical names such as tetrahydrocannabinol and cannabidiol. Following the initial query, we identified unanticipated variants, including misspellings, abbreviations, chemical isomers, and informal or international brand names. Considering sensitivity and specificity, each term was manually reviewed and either excluded (unrelated or ambiguous) or mapped to an exposure group. The details for this process are included in an R script and a table of the term mappings is provided in Online Appendix 1.

**FIGURE 1:**
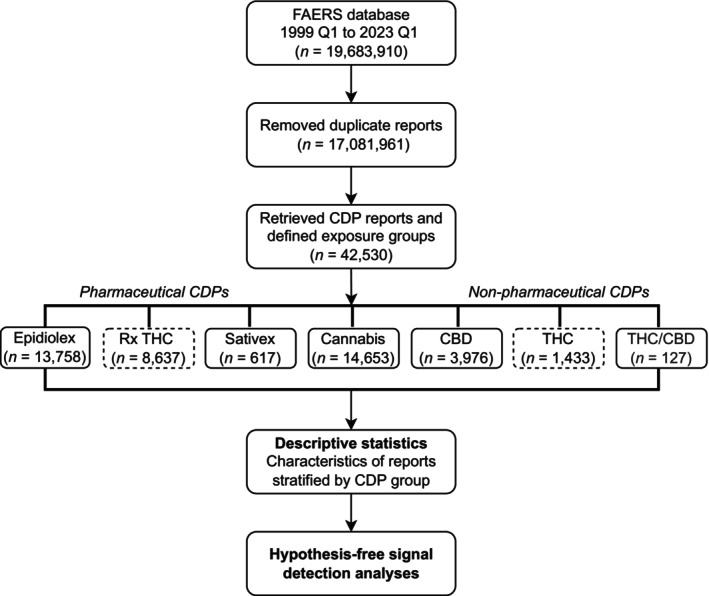
Flowchart of CDP exposure grouping and analyses in FAERS (1999 Q1 to 2023 Q1). After deduplication, we retrieved FAERS reports containing CDP‐related terms and grouped them into seven exposure categories: Pharmaceuticals (Epidiolex, Rx THC, Sativex) and non‐pharmaceuticals (Cannabis, CBD, THC, THC/CBD). We computed descriptive statistics by exposure group and conducted hypothesis‐free disproportionality analyses for Epidiolex and non‐pharmaceutical CBD. CBD, Cannabidiol; CDP, Cannabis‐derived product; FAERS, US Food and Drug Administration Adverse Event Reporting System; Rx THC, Pharmaceutical‐containing THC; THC, Non‐pharmaceutical containing tetrahydrocannabinol.

To identify potential toxicity to chemical constituents of cannabis products, we manually classified CDP reports on active ingredient and product quality control standards—similar to grouping pharmaceuticals by therapeutic class. Pharmaceutical CDPs containing derived tetrahydrocannabinol (THC), such as dronabinol (Marinol and Syndros) and nabilone (Cesamet), were grouped under prescription THC: (‘Rx THC’) (Figure 1). Reports with the brand name Epidiolex or Epidyolex were grouped as ‘Epidiolex’, and those referring exclusively to nabiximols (Sativex) were grouped as ‘Sativex’. Among non‐pharmaceutical CDPs, we categorized delta‐8‐tetrahydrocannabinol (D8‐THC) and delta‐9‐tetrahydrocannabinol (D9‐THC) together as ‘THC’, while the ‘CBD’ group included terms mentioning cannabidiol without reference to the branded product. Reports mentioning both THC and CBD were grouped under ‘THC/CBD’. Finally, reports with no reference to a particular cannabinoid were classified under the ‘Cannabis’ group and included terms such as cannabis, cannabinoids, or marijuana. The complete list of CDP terminology is provided in the Online Appendix 1.

### 2.3 Outcome Definition

Given the hypothesis‐free design, no AEs were specified a priori, and MedDRA terms were included at the PT level using version 27.0. Serious outcomes were summarized descriptively to characterize the severity of reports.

### 2.4 Calendar Time Restrictions

For the descriptive analysis, we included reports from 1999 Q1 to 2023 Q1 to capture the early expansion of medical cannabis legalization in the US [16]. For the disproportionality analyses, we restricted the study period to include 2018 Q2 to 2023 Q1 to reflect the later approval of Epidiolex by the FDA and legalization of hemp‐derived CBD with the 2018 *Farm Bill* [17].

### 2.5 Comparators

Given the lack of an a priori set of AEs, we included all other drugs reported in FAERS, while excluding the CDP of interest, as the comparator set.

### 2.6 Statistical Analysis

For the descriptive analysis, we summarized report characteristics, including age, sex, reporter country, role of the CDP in the report, and reported outcomes. Descriptive statistics were stratified by cannabinoid group, as defined through the mapping exercise. Reports with missing data for a variable were categorized as ‘missing’.

We estimated signals of disproportionate reporting for Epidiolex and non‐pharmaceutical CBD using three algorithms: the proportional reporting ratio (PRR) [18], the reporting odds ratio (ROR) [19], and the Bayesian confidence propagation neural network (BCPNN) [20], which estimates the information component (IC). The PRR is a reporting‐based analogue to relative risk and the ROR is the reporting version of an odds ratio. The IC compares observed drug‐AE reports against their expectation under independence and a Bayesian framework ‘shrinks’ estimates using a prior of independent reporting, reducing false positive rates in small sample settings—a known concern with PRR and ROR [20]. A signal was flagged for PRR and ROR if the lower bound of a 95% confidence interval (CI) of the point estimate exceeded 1, and for IC, the lower bound of a 95% credible interval (CrI) exceeded 0. All analyses were done with R version 4.2.3.

## 3 Results

### 3.1 CDP Terminology and Reporting Patterns

Of the 17 081 961 reports after data cleaning, we identified 42 530 reports that included a CDP‐related term, yielding 1204 unique terms. We excluded 44 related to minor cannabinoids or THC synthetics (e.g., ‘cannabinol’ and ‘spice/K2’, respectively). The largest categories by number of terms were CBD (339; 29.2%), Cannabis (319; 27.5%), and THC (244; 21.0%), while Epidiolex had the fewest (14; 1.2%) (Online Appendix 1).

Report frequencies by category differed from the corresponding distribution of terms (Table 1). The most frequently reported CDP was Cannabis (*n* = 14 653; 33.9% of CDP reports) and Epidiolex (*n* = 13 758; 31.8%), while Sativex (*n* = 617; 1.4%) and THC/CBD (*n* = 127; 0.3%) were less representative. Overall, pharmaceutical CDPs (Rx THC, Epidiolex, and Sativex) accounted for 53.3% of the CDP reports, while non‐pharmaceuticals (THC, THC/CBD, CBD, and Cannabis) constituted 46.7%.

**TABLE 1:** Patient demographics characteristics with reported use of CDPs queried from FAERS between 1999 Q1 and 2023 Q1.

Characteristic	Cannabis‐derived products unique reports (*n* = 42 530)						
	Pharmaceutical			Non‐pharmaceutical			
	Rx THC (*n* = 8637)	Epidiolex (*n* = 13 758)	Sativex (*n* = 617)	THC (*n* = 1433)	THC/CBD (*n* = 127)	CBD (*n* = 3976)	Cannabis (*n* = 14 653)
Age in years^a^, *n* (%)							
0–12	118 (1.4)	638 (4.6)	4 (0.6)	16 (1.1)	5 (3.9)	328 (8.2)	155 (1.1)
13–17	201 (2.3)	230 (1.7)	0 (0)	61 (4.3)	4 (3.1)	96 (2.4)	856 (5.8)
18–35	764 (8.8)	424 (3.1)	54 (8.8)	605 (42.2)	29 (22.8)	395 (9.9)	4713 (32.2)
36–54	1695 (19.6)	180 (1.3)	328 (53.2)	395 (27.6)	41 (32.3)	698 (17.6)	3437 (23.5)
55–69	1970 (22.8)	72 (0.5)	123 (19.9)	115 (8.0)	19 (15.0)	684 (17.2)	1666 (11.4)
70+	1249 (14.5)	17 (0.1)	16 (2.6)	34 (2.4)	8 (6.3)	475 (11.9)	515 (3.5)
Missing	2640 (30.6)	12 197 (88.7)	92 (14.9)	207 (14.4)	21 (16.5)	1300 (32.7)	3311 (22.6)
Sex, *n* (%)							
Female	4190 (48.5)	1678 (12.2)	394 (63.9)	524 (36.6)	69 (54.3)	2131 (53.6)	5520 (37.7)
Male	3918 (45.4)	1990 (14.5)	204 (33.1)	762 (53.2)	49 (38.6)	1234 (31.0)	7996 (54.6)
Missing	529 (6.1)	10 090 (73.3)	19 (3.1)	147 (10.3)	9 (7.1)	611 (15.4)	1137 (7.8)
Role, *n* (%)							
Primary suspect drug	775 (9.0)	12 788 (92.9)	7 (1.1)	346 (24.1)	61 (48.0)	353 (8.9)	78 (0.5)
Secondary suspect drug	649 (7.5)	157 (1.1)	68 (11.0)	736 (51.4)	16 (12.6)	637 (16.0)	7656 (52.2)
Interacting drug	55 (0.6)	40 (0.3)	5 (0.8)	54 (3.8)	6 (4.7)	148 (3.7)	189 (1.3)
Concomitant drug	7158 (82.9)	773 (5.6)	537 (87.0)	297 (20.7)	44 (34.6)	2838 (71.4)	6730 (45.9)
Outcome, *n* (%)							
Hospitalization	3459 (36.7)	2942 (42.5)	281 (42.6)	409 (22.0)	46 (30.3)	915 (25.6)	4703 (25.8)
Life‐threatening	338 (3.6)	47 (0.7)	32 (4.8)	105 (5.6)	16 (10.5)	123 (3.4)	650 (3.6)
Death	1527 (16.2)	837 (12.1)	17 (2.6)	526 (28.3)	1 (0.7)	188 (5.3)	3869 (21.2)
Other	3839 (40.7)	3056 (44.2)	308 (46.7)	650 (34.9)	59 (38.8)	2123 (59.5)	8363 (45.8)
Disability	188 (2.0)	27 (0.4)	22 (3.3)	54 (2.9)	11 (7.2)	156 (4.4)	441 (2.4)
Congenital abnormality	20 (0.2)	5 (0.1)	0 (0)	9 (0.5)	0 (0)	4 (0.1)	152 (0.8)
Required intervention	58 (0.6)	4 (0.1)	0 (0)	107 (5.8)	19 (12.5)	59 (1.7)	79 (0.4)
Reporting country, *n* (%)							
Canada	1426 (16.5)	12 (0.1)	22 (3.6)	59 (4.1)	20 (15.7)	793 (19.9)	2359 (16.1)
United States	6066 (70.2)	13 355 (97.1)	9 (1.5)	803 (56.0)	93 (73.2)	2407 (60.5)	7446 (50.8)
Europe	675 (7.8)	219 (1.6)	571 (92.5)	358 (25.0)	13 (10.2)	581 (14.6)	3745 (25.6)
Other	45 (0.5)	125 (0.9)	12 (1.9)	104 (7.3)	1 (0.8)	130 (3.3)	425 (2.9)
Missing	425 (4.9)	47 (0.3)	3 (0.5)	109 (7.6)	0 (0)	65 (1.6)	678 (4.6)

*Note:* The estimated numbers of CDP reports are not mutually exclusive because people could have reported more than one suspected CDP in FAERS. Percentages were calculated using the total number of reports for each specific CDP group as the denominator.

Abbreviations: CBD, cannabidiol; CDP, cannabis‐derived product; FAERS, US Food and Drug Administration Adverse Event Reporting System; Rx THC, pharmaceutical‐containing THC; THC, non‐pharmaceutical containing tetrahydrocannabinol.

^a^ Age stratifications commonly used in cannabis epidemiology and public health surveillance [8, 26].

Demographic characteristics differed with reports for CBD, Rx THC, and Sativex more frequently associated with females aged 36–69, whereas THC and Cannabis involved males aged 18–54 years. Epidiolex was the primary suspect drug in 92.9% of reports (*n* = 12 788), highest among all CDPs, while Cannabis was the lowest, with 0.5% of reports. Reports for CBD, Rx THC, and Sativex were often flagged as concomitant drugs, while THC and Cannabis were commonly reported as secondary suspects. Age was frequently missing, but when available, children aged 0–12 years accounted for 8.2% of CBD and 4.6% of Epidiolex reports, the highest among CDPs. Serious outcomes were more frequently reported for THC and Cannabis, with death recorded in 28.3% of THC and 23.3% of Cannabis reports, although these proportions do not imply causality.

### 3.2 Hypothesis‐Free Signal Detection of CBD‐Containing Products

Given the propensity for false positives expected with PRR and ROR [21], and the absence of a priori AE selection, we used IC as the primary method to identify potential signals (Table 2). In the disproportionality analysis for Epidiolex, potential signals were primarily with nervous system disorders (18.75%), psychiatric disorders (16.48%), and injury, poisoning and procedural complications (10.23%). Seizure‐related AEs comprised a proportion of the strongest signals: seizure cluster (IC, 6.36; 95% CrI 6.05–6.68), change in seizure presentation (IC, 6.07; 95% CrI 5.68–6.45), atonic seizures (IC, 6.02; 95% CrI 5.64–6.41), seizure (IC, 5.47; 95% CrI 5.43–5.51), and weight abnormal (IC, 5.67; 95% CrI 5.40–5.93) (Table 2). CBD signals were classified under nervous system disorders (13.79%), investigations (11.13%), and general disorders (10.03%), with the highest linked to neoplasm‐related events (IC range: 3.5–5.1) (Table 2). The strongest signals for CBD included multiple‐drug resistance (IC, 5.73; 95% CrI 5.35–6.12), blood pressure diastolic decreased (IC, 4.88; 95% CrI 4.49–5.27), device related thrombosis (IC, 5.00; 95% CrI 4.36–5.65), malignant cranial nerve neoplasm (IC, 5.1; 95% CrI 4.33–5.87), and retro‐orbital neoplasm (IC, 5.08; 95% CrI 4.31–5.85) (Table 2). Noting the seizure‐related indication for Epidiolex we ran post hoc subgroup analyses stratified by this indication and presented the results in Online Appendix 3.

**TABLE 2:** The top IC estimates for Epidiolex (top panel) and CBD (bottom panel) at the preferred term level (FAERS 2018 Q2 to 2023 Q1).

	Epidiolex IC (95% CrI)	CBD IC (95% CrI)
Top 15 PTs by Epidiolex disproportionality		
Seizure cluster	6.36 (6.05 to 6.68)	2.43 (0.44 to 4.42)
Change in seizure presentation	6.07 (5.68 to 6.45)	2.88 (1.19 to 4.57)
Atonic seizures	6.02 (5.64 to 6.41)	2.51 (0.52 to 4.5)
Seizure	5.47 (5.43 to 5.51)	2.89 (2.68 to 3.1)
Weight abnormal	5.67 (5.40 to 5.93)	NSD
Product supply issue	5.31 (5.14 to 5.48)	NSD
Anticonvulsant drug level increased	5.56 (5.14 to 5.99)	2.80 (1.12 to 4.49)
Emergency care	5.39 (5.12 to 5.65)	NSD
Product administration interrupted	4.97 (4.77 to 5.18)	1.80 (0.56 to 3.03)
Sudden unexplained death in epilepsy	4.66 (3.95 to 5.36)	3.47 (2.13 to 4.82)
Drooling	4.20 (3.86 to 4.54)	1.76 (0.27 to 3.25)
Prescribed overdose	3.94 (3.72 to 4.16)	NSD
Product distribution issue	3.93 (3.59 to 4.27)	NSD
Therapy responder	4.45 (3.56 to 5.33)	NSD
Generalized tonic–clonic seizure	3.78 (3.55 to 4.01)	3.06 (2.51 to 3.60)
Top 15 PTs by CBD disproportionality		
Multiple‐drug resistance	NSD	5.73 (5.35 to 6.12)
Blood pressure diastolic decreased	NSD	4.88 (4.49 to 5.27)
Device related thrombosis	NSD	5.00 (4.36 to 5.65)
Malignant cranial nerve neoplasm	NSD	5.10 (4.33 to 5.87)
Retro‐orbital neoplasm	NSD	5.08 (4.31 to 5.85)
Neuroblastoma recurrent	NSD	4.96 (4.17 to 5.76)
Blood pressure diastolic abnormal	NSD	4.65 (4.12 to 5.18)
Tonic convulsion	3.94 (3.19 to 4.69)	4.81 (4.09 to 5.54)
Metal poisoning	NSD	4.78 (3.96 to 5.60)
Urine leukocyte esterase positive	NSD	4.42 (3.60 to 5.24)
Behavior disorder	3.01 (2.57 to 3.46)	4.08 (3.54 to 4.61)
Sinus headache	NSD	4.18 (3.49 to 4.87)
Blood pressure systolic abnormal	NSD	4.12 (3.45 to 4.79)
Blood pressure systolic increased	NSD	3.76 (3.38 to 4.14)
Post viral fatigue syndrome	NSD	4.33 (3.36 to 5.30)

Abbreviations: CrI, credible interval; FAERS, US FDA Adverse Event Reporting System; IC, information component; PT, preferred term; NSD, no signal detected (lower bound of the 95% credible interval for the information component IC_025_ ≤ 0).

**FIGURE 2:**
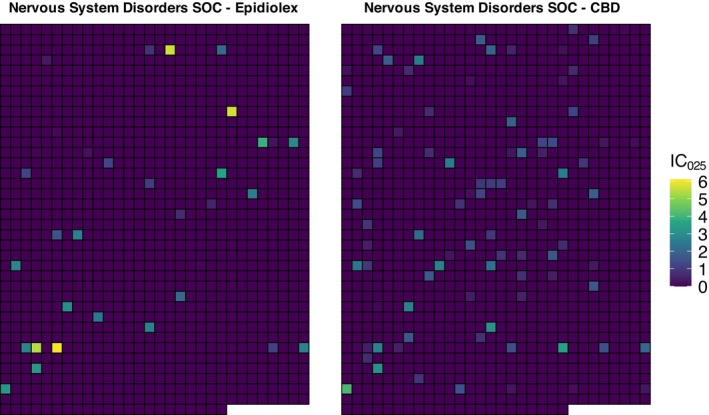
Sector maps of signals identified in the nervous system disorders SOC for Epidiolex (left) and CBD (right). Each square represents a MedDRA PT within the nervous system disorders SOC. PT positions are identical across maps. Colors represent the lower bound of the 95% credible interval for the information component (IC_025_): Darker shades indicate IC_025_ ≤ 0 (no signal), and brighter shades indicate IC_025_ > 0 (higher‐than‐expected reporting for the drug‐event pair). CBD, Cannabidiol; IC_025_, 95% credible interval for the information component; PT, Preferred term; SOC, System organ class.

For both Epidiolex and CBD, the highest IC estimates were classified under nervous system disorders SOC, although the nature of signals differed between groups. We present IC estimates in a sector map (Figure 2) comparing pharmaceutical and non‐pharmaceutical CBD, where each cell corresponds to the same PT in the nervous system SOC. The color gradient from dark purple to bright yellow represents the lower bound of the 95% CrI for the IC (IC_025_), with darker shades indicating IC_025_ ≤ 0 (no signal) and brighter shades indicating IC_025_ > 0. We observed 33 highlighted cells for Epidiolex and 88 for CBD. Of the 33, 13 (39.4%) contained seizure or convulsion, with ICs ranging from 1.62 to 6.36. In contrast, CBD reports showed 2.6‐fold more signals; however, only 12 (13.6%) were seizure/convulsion related, with ICs ranging from 1.97 to 4.81. Most CBD‐related signals in this SOC were linked to diverse conditions, such as multiple sclerosis, Parkinson's, and neuralgia.

## 4 Discussion

Our study evaluated the feasibility of using the FAERS database for cannabis‐related safety surveillance. We grouped diverse CDP terminologies into relevant categories, stratified by pharmaceutical and recreational motivations for utilization, and descriptively summarized reporting characteristics across groups. We conducted hypothesis‐free disproportionality analysis of Epidiolex and non‐pharmaceutical CBD as a relative comparison of signal detection profiles for the same cannabinoid under different regulatory and utilization contexts to assess feasibility. We observed substantial heterogeneity in CDP reporting terminology, patterns, and signal detection results, which was also observed in a post hoc subgroup analysis stratified by seizure indication.

We identified 1160 terms describing CDPs, with fragmented and inconsistent nomenclature. Terminology included diversity of products, mentioning legal statuses (e.g., ‘illegal marijuana’ and ‘legal cannabis’), highlighting the additional complexity of studying CDPs in FAERS beyond the usual concerns of linking commercial and chemical terms with small proportions of spelling errors [22]. While we provide a preliminary effort for categorizing CDP terms into meaningful groupings for future research, we demonstrated that manual intervention and contextual standardization are additional necessary steps for data cleaning in CDP‐related signal detection analysis. These processes are more complex and time‐consuming than for pharmaceutical drugs, but the risk of information bias and exposure misclassification may further compromise the reliability of safety signal detection [23] and we have provided our mappings to support implementation in future studies in Online Appendix 1.

Despite these challenges, our descriptive analysis revealed reporting patterns consistent with known CDP indications and user demographics. Epidiolex reports were predominantly flagged as primary suspect and frequently involved pediatric patients, reflecting its approved indication for refractory epilepsy [1]. In contrast, non‐pharmaceutical CBD reports were more heterogeneous, with higher representation of women and older adults, and concomitant reporting alongside other products. This pattern may reflect broader use of CBD formulations for symptom management in conditions such as chronic pain, anxiety, and sleep disturbances, consistent with previous surveys and observational studies [24, 25].

Reports involving THC and Cannabis were associated with males aged 18 to 54 years and were often recorded as secondary suspects. This reflects national cannabis surveys, where males report higher percentages of cannabis use than females [26, 27]. Our findings highlighted that, while Epidiolex and non‐pharmaceutical CBD both contain cannabidiol as a primary active ingredient, they likely differ in composition, purity, and concentration. Moreover, reporters vary in utilization and demographic characteristics, which may introduce heterogeneity in motivation to report, impacting the nature of reporting bias.

The disproportionality analysis highlighted these differences as two distinct signal detection profiles were observed. Epidiolex exhibited strong signals for seizure‐related adverse events, reflecting indication and heightened reporting in clinical settings. Such findings were anticipated, as pharmaceutical drugs are subject to legislative requirements for mandatory reporting [28]. In contrast, non‐pharmaceutical CBD reports showed a broader spectrum of signals, including neoplasm events and neurological disorders, likely reflecting the use of CBD to treat or manage these symptoms or health conditions. Seizure‐related events were less prominent compared to Epidiolex reports. The indication‐specific reporting profiles were noted further in our subgroup analyses (Online Appendix 3) where, notably, in the non‐seizure subgroup, the top signals detected for the non‐pharmaceutical version of CBD had no counterparts in the Epidiolex analysis with one exception (behavior disorder).

While some signals reflect ADRs consistent with the Epidiolex product monograph [1] (e.g., drooling, sedation, fluctuations in weight), observed differences with non‐pharmaceutical CBD imply factors beyond indication alone. Reporting context, product composition, dose, product quality, and patient characteristics may influence reporting behaviors [29]. Reporting bias and attribution effects may contribute to non‐pharmaceutical CBD signals, as individuals without medical supervision may be less likely to recognize or report AEs, or may misattribute them to underlying conditions [30]. In contrast, patients treated with Epidiolex are monitored in specialized care settings, where AEs are systematically documented, increasing the capture of seizure‐related events and other known risks. Selection bias may contribute, as individuals with chronic or severe illnesses may proactively report AEs, particularly when concerns are overlooked by clinicians [31]. Furthermore, non‐pharmaceutical CBD formulations exhibit variability in composition, purity, and labeling accuracy, potentially contributing to ADRs themselves as well as to reports linked to contaminants or inconsistent cannabinoid content [32]. Therefore, differences in regulatory status, prescribing practices, utilization patterns, and reporting behaviors may influence the signals detected for the same cannabinoid.

Our study has limitations. First, grouping CDP terms relied on manual, subjective decision‐making based on reported terms and associated cannabinoid pharmacology and regulatory status. Although this process was designed to maximize sensitivity and specificity, it may have introduced some degree of exposure misclassification, particularly for ambiguous or inconsistently reported terms. Second, our analysis was a broad, hypothesis‐free screening approach intended to compare high‐level signal detection profiles between pharmaceutical and non‐pharmaceutical CBD, rather than a study robustly designed to identify or validate individual ADRs. Hence, the observed signals may be influenced by systematic biases inherent to spontaneous reporting, such as confounding by association, competition bias, and under‐reporting or stimulated reporting [33, 34, 35], that were not addressed given the exploratory nature of this design. The choice of comparators in disproportionality analysis has been shown to influence both the magnitude and directionality of signal estimates [36, 37], and we used the full‐data reference set, as this approach was not intended to optimize signal detection for any single ADR.

Accordingly, signals identified should not be interpreted as definitive evidence of the CDP safety profile or to infer differences between pharmaceutical and non‐pharmaceutical formulations. They reflect CDP reporting patterns within a specific timeframe and context. These findings are exploratory, and individual drug‐event combinations require validation in rigorously designed studies. Finally, disproportionality estimates alone cannot establish causality and must be evaluated in conjunction with complementary sources of evidence, including biological plausibility, controlled clinical data, patient‐level characteristics, and alternative explanations for the reported events [38].

## 5 Conclusion

Our feasibility study showed that FAERS may provide a valuable foundation for hypothesis generation in cannabis pharmacovigilance, but there are challenges beyond standard limitations of disproportionality analysis. Inconsistent terminology leading to ambiguous exposure definitions, the potential for specific forms of reporting bias arising from differing patterns of utilization for the same cannabinoid, and confounding by indication stratified by regulatory status contribute to the uncertainty underlying CDP signal detection. These limitations can substantially influence disproportionality estimates and complicate interpretation, and the informativeness of such estimates in isolation remains limited without more targeted study designs. Nevertheless, our findings suggest that rigorous methodological design, including population‐specific analytical approaches and systematic evaluation of exposure classification, can improve the reliability of CDP safety surveillance; however, future work is needed to evaluate their impacts. We posit that coordinated efforts by regulatory authorities, independent researchers, and pharmacovigilance experts to develop standardized nomenclature and operational definitions for CDPs may contribute to a more rigorous and transparent evaluation of the safety of cannabis‐derived therapies.

## Author Contributions

**Priscilla O. M. V. Lopes:** conceptualization, formal analysis, visualization, writing – original draft, and writing – review and editing; **Cory S. Harris:** conceptualization, funding acquisition, methodology, and writing – review and editing; **Christopher A. Gravel:** conceptualization, methodology, writing – review and editing, and supervision. All authors read and approved the final version.

## Funding

This study was supported by the Natural Sciences and Engineering Research Council of Canada (NSERC) CREATE Quality Assurance and Quality Control for Cannabis Production, Products and Training Program, grant number 543319‐2020.

## Ethics Statement

The authors have nothing to report.

## Consent

The authors have nothing to report.

## Conflicts of Interest

C.S.H. has received research support from companies in the cannabis industry for unrelated projects including chemical analysis, process optimization, and new product development. The other authors declare no conflicts of interest.

## Supporting information


**Appendix 1** Cannabis‐derived product terminology inventory.
**Appendix 2**. The top 15 disproportionality analysis estimates for Epidiolex (top panel) and CBD (bottom panel) at the preferred term level, ranked by IC_025_ (FAERS Q2 2018 to Q1 2023).
**Appendix 3A**. The top 30 disproportionality analysis estimates for Epidiolex (top panel) and CBD (bottom panel) with seizure indication at the preferred term level, ranked by IC_025_ (FAERS Q2 2018 to Q1 2023).
**Appendix 3B**. The top 30 disproportionality analysis estimates for Epidiolex (top panel) and CBD (bottom panel) without seizure indication, at the preferred term level, ranked by IC_025_ (FAERS Q2 2018 to Q1 2023).
**Appendix 4**. R code for CDP Terminology Identification in FAERS.

## Data Availability

The raw data used for the analyses are publicly available here: https://fis.fda.gov/extensions/FPD‐QDE‐FAERS/FPD‐QDE‐FAERS.html. The code can be requested from Priscilla O. M. V. Lopes.
