# Supplementary material for: A Feasibility Assessment of the FDA Adverse Event Reporting System for the Detection of Cannabis‐Related Safety Signals
Source: Pharmacoepidemiol Drug Saf. 2026 May 13;35:e70392. doi: 10.1002/pds.70392 (PMC13172948; doi:10.1002/pds.70392)
Supplement: Supplementary file 1 — Appendix 1 Cannabis‐derived product terminology inventory. Appendix 2. The top 15 disproportionality analysis estimates for Epidiolex (top panel) and CBD (bottom panel) at the preferred term level, ranked by IC025 (FAERS Q2 2018 to Q1 2023). Appendix 3A. The top 30 disproportionality analysis estimates for Epidiolex (top panel) and CBD (bottom panel) with seizure indication at the preferred term level, ranked by IC025 (FAERS Q2 2018 to Q1 2023). Appendix 3B. The top 30 disproportionality analysis estimates for Epidiolex (top panel) and CBD (bottom panel) without seizure indication, at the preferred term level, ranked by IC025 (FAERS Q2 2018 to Q1 2023). Appendix 4. R code for CDP Terminology Identification in FAERS. [file PDS-35-e70392-s001.docx]

**Running Title: Cannabis Safety Signal Detection**

**A Feasibility Assessment of the FDA Adverse Event Reporting System for the Detection of Cannabis-Related Safety Signals**

**Authors:** Priscilla O. M. V. Lopes^1,2^, Cory S. Harris^2,3^, Christopher A. Gravel^1,4,5^

**Affiliations:**

1. School of Epidemiology and Public Health, University of Ottawa, Ottawa, OΝ, Canada
2. Department of Biology, University of Ottawa, Ottawa, ON, Canada
3. Department of Chemistry and Biomolecular Sciences, University of Ottawa, Ottawa, ON, Canada
4. Department of Mathematics and Statistics, University of Ottawa, Ottawa, ON, Canada
5. Data Literacy Research Institute, University of Ottawa, Ottawa, ON, Canada

**Corresponding author:**

Christopher A. Gravel, PhD

Assistant Professor

School of Epidemiology and Public Health, University of Ottawa

600 Peter Morand, 114 C

Ottawa, Ontario, Canada K1G5Z3

613-562-5800 (3062)

[cgravel@uottawa.ca](file:///Users/priscillalopes/Library/Mobile%20Documents/com~apple~CloudDocs/MSc%20Epi/Manuscripts/Feasibility%20Assessment/PDS/cgravel@uottawa.ca)

**Online Appendix 1** Cannabis-derived product terminology inventory

Lists the search/aggregation terms used to classify FAERS *drugname* entries into seven CDP groups (Rx THC, Sativex, Epidiolex, THC, THC/CBD, CBD, and Cannabis). Terms include brand/generic names, common synonyms, spelling variants, and dosage-form indications. Grouping prioritizes regulatory status (pharmaceutical vs non-pharmaceutical) and composition (single-agent vs fixed THC:CBD combinations). Minor cannabinoids (e.g., cannabinol) and synthetic cannabinoids (e.g., “spice/K2”) were excluded.

The process for generating the mappings was done by string matching using 14 predefined terms: "cannab", "canab", "mariju", "marih", "thc", "cbd", "nabixi", "dronab", “nabilo", "cesam", "syndros", "marino", "epidiol", "sative", followed by manual review by two of the authors (POMVL and CSH). A short script is provided in the online appendix to demonstrate the process.

Abbreviations: CBD: cannabidiol; CDP, cannabis-derived product; FAERS: U.S. FDA Adverse Event Reporting System; Rx THC: pharmaceutical-containing THC; THC: non-pharmaceutical containing tetrahydrocannabinol

**Rx THC Terminology**

| **term** | **group** |
| --- | --- |
| MARINOL /00897601/ | Rx THC |
| MARINOL | Rx THC |
| Marinol | Rx THC |
| DRONABINOL | Rx THC |
| MARINOL (UNITED STATES) | Rx THC |
| CESAMET | Rx THC |
| DRONABINOL. | Rx THC |
| MARINOL (DRONABINOL) | Rx THC |
| NABILONE | Rx THC |
| MARTINOL (DRONABINOL) (DRONABINOL) | Rx THC |
| MARINOL /00003301/ | Rx THC |
| MARINOL (DRONABINOL) (2.5 MILLIGRAM, CAPSULES) | Rx THC |
| DRONABINOL (DRONABINOL) (UNKNOWN) | Rx THC |
| MARINOL (DRONABINOL) (UNKNOWN) | Rx THC |
| DRONABINOL (DRONABINOL) (10 MILLIGRAM, CAPSULES) | Rx THC |
| DRONABINOL (DRONABINOL) (CAPSULES) | Rx THC |
| DRONABINOL(DRONABINOL) | Rx THC |
| DRONABINOL(DRONABINOL)(UNKNOWN) | Rx THC |
| dronabinol | Rx THC |
| nabilone | Rx THC |
| DRONABINOOL | Rx THC |
| DRONABINOL CAPSULES | Rx THC |
| CESAMET (NABILONE) | Rx THC |
| marinol | Rx THC |
| MARINIOL (DRONABINOL) | Rx THC |
| DRONABINOL (DRONABINOL) | Rx THC |
| MARINOL (DRONABINOL) (DRONABINOL) | Rx THC |
| MARINOL (mecobalamin) | Rx THC |
| Dronabinol | Rx THC |
| MARINOL (dronabinol) | Rx THC |
| Dronabinol Capsules | Rx THC |
| MARINOL /00897601/ | Rx THC |
| MARINOL (DRONABINOL) (CAPSULES) | Rx THC |
| MARIINOL (DRONABINOL) | Rx THC |
| DRONABINOL (UNKNOWN) | Rx THC |
| DRONABINOL CAPSULES USP | Rx THC |
| Marinol/Dronabinol/THC | Rx THC |
| Nabilone | Rx THC |
| Dronabinol (THC) | Rx THC |
| Dronabinol Softgel Caps | Rx THC |
| MARINOL(DRONABINOL) | Rx THC |
| MARINOL 2.5MG | Rx THC |
| MARINOL (DRONABINOL) (CAPSULES) (DRONABINOL) | Rx THC |
| MARINOL (UNITED STATES) (DRONABINOL) | Rx THC |
| DRONABINOL (MEDICAL MARIJUANA) | Rx THC |
| DRONABINOL (MARINOL) | Rx THC |
| DRONABINOL 2.5MG | Rx THC |
| MARINOL [ALLOPURINOL] | Rx THC |
| DRONABINOL Capsule | Rx THC |
| MARINOL /00003301/ | Rx THC |
| DRONABINOL (IMARINOL) | Rx THC |
| DRONABINAL | Rx THC |
| DRONABINOL 5MG | Rx THC |
| Marinot | Rx THC |
| NABILONE 250 MCG | Rx THC |
| DRONABINOL (MARINOL PO) | Rx THC |
| DRONABIHOL | Rx THC |
| RAN-NABILONE | Rx THC |
| MARINOL (ALLOPURINOL) | Rx THC |
| Dronabinol w/ Aspirin | Rx THC |
| MARINOL CAP | Rx THC |
| DRONABINAL (MARINAL) | Rx THC |
| DRONABINOL (MSTINOL) | Rx THC |
| DRONABINOL CAPSULE, USP CIII | Rx THC |
| TEVA NABILONE | Rx THC |
| Dronabinol Capsule, USP CIII | Rx THC |
| SYNDROS | Rx THC |
| MARINORL | Rx THC |
| NIDA CANNABIS/DRONABINOL | Rx THC |
| DRONABI NCL | Rx THC |
| NABILON | Rx THC |
| Marinol 5 mg. | Rx THC |
| Dronabinoloel | Rx THC |
| Dronabinol 5mg | Rx THC |
| Dronabinol j5mg | Rx THC |
| DRONABINOL CAP 10MG | Rx THC |
| dronabinol 2.5 | Rx THC |
| MARINOL [DRONABINOL] | Rx THC |
| dronabinol cap 10mg | Rx THC |
| NABILONE CAPSULE | Rx THC |
| NABILONE CAPSULES | Rx THC |
| DRONABINOL CAP 2.5MG | Rx THC |
| DRONABINOL 2.5 MG CAPSULE CIII | Rx THC |
| Dronabinol, Dulera, Montelukast | Rx THC |
| dronabinole | Rx THC |
| NABILONE MYLAN | Rx THC |
| DRONABOL | Rx THC |
| Dronabinol capsules | Rx THC |
| DRONABINOL CAPSULES, USP 2.5 MG. RX ONLY | Rx THC |
| dronabinal cap 5mg | Rx THC |
| MARINOL [MECOBALAMIN] | Rx THC |
| BEDIOL (CANNABIDOL\DRONABINOL) | Rx THC |
| dronabinol 2.5 mg | Rx THC |
| marinol cap 5 mg | Rx THC |
| dronabinol 2.5mg | Rx THC |
| DRONABINOL 5MG CAPSULES | Rx THC |
| MARINOL CAP 5MG | Rx THC |
| Dronabinol 2.5 mg, oral | Rx THC |
| Dronabinol 2.5mg | Rx THC |
| DRONABINOL (DRONABINOL 2.5MG CAP) | Rx THC |
| Marinol 2.5mg | Rx THC |
| DRONABINOL 5 MG | Rx THC |
| DRONABINOL CAPSULE CIII | Rx THC |
| MARINOL 5MG | Rx THC |
| DRONABINOL 10MG | Rx THC |
| MARINOL 10MG | Rx THC |
| DRONABINOL 2.5MG CAPSULES | Rx THC |
| Marinol 10mg | Rx THC |
| Dronabinol 2.5MG capsules | Rx THC |
| ^MARINOL^ | Rx THC |
| dronabinol 5?10 | Rx THC |
| DARUNAVIR , GENERIC FOR MARINOL | Rx THC |
| Dronabinol 5MG capsules | Rx THC |
| THC (DRONABINOL) | Rx THC |
| DRONABINOL (Medical Marijuana) | Rx THC |
| MARINOL OMEGA 3 | Rx THC |
| dronabinol 5 mg | Rx THC |
| RAN NABILONE | Rx THC |
| Marinol 5mg | Rx THC |
| NABILONA | Rx THC |
| Dronabino | Rx THC |
| Dronabinol 5mg BID | Rx THC |
| Cetirizine DronabinoL | Rx THC |
| MARINOL 10 MG | Rx THC |
| Dronabinol 2.5 mg | Rx THC |
| DRONABIN OL | Rx THC |
| Dronabinol Oral Capsule 2.5 MG | Rx THC |
| Marinol 5 mg | Rx THC |
| DRONABINOL CAP 5MG | Rx THC |
| MAG-OXIDE MARINOL | Rx THC |
| MARINOL (DRONABINOL (DELTA-9- TETRAHYDROCANNABINOL)) | Rx THC |
| COMPASSIA (DRONABINOL) | Rx THC |
| NABILONE (NABILONE) | Rx THC |
| MARINOL (ALGAE/CALCIUM PHOSPHATE, MONOBASIC/IODINE NOS/PHOSPHORIC ACID | Rx THC |
| DRONABINOL 2.5 MG | Rx THC |
| NARINOL (DRONABINOL) | Rx THC |
| MARIOL (DRONABINOL) | Rx THC |
| DRONABINOAL | Rx THC |
| MARNOL (DRONABINOL) | Rx THC |
| MARINOL (DRONABINAOL) | Rx THC |
| MARINOL TABLETS | Rx THC |
| MARINOL 2.5 | Rx THC |
| MARINOL (DRONABINOL) TABLET | Rx THC |
| MARINOL (DRONABINOL),(2.5 MILLIGRAM, CAPSULES) | Rx THC |
| MARINOL (BRONABINOL) | Rx THC |
| MARINOL (DRONABINOL) (10 MILLIGRAM, CAPSULES) | Rx THC |
| NABILONE (CESAMET) | Rx THC |
| MARINOL (*ALGAE/*ALLOPURINOL/*CALCIUM PHOSPHATE, MONOBASIC/*DROABINNOL | Rx THC |
| MARINOL (ALGAE/ALLOPURINOL/CALCIUM PHOSPHATE MONOBASIC/*DRONABINOL/*IO | Rx THC |
| MARINOL(ALGAE/ALLOPURINOL/CALCIUM PHOSPHATE | Rx THC |
| (NABILONE) | Rx THC |
| DRONABINOL 2.5 G | Rx THC |
| DRONABIONL | Rx THC |
| DELTA(9)- TETRAHYDROCANNABINOL (DRONABINOL) | Rx THC |

**Sativex Terminology**

| **term** | **group** |
| --- | --- |
| SATIVEX SPRAY | Sativex |
| SATIVEX | Sativex |
| NABIXIMOLS | Sativex |
| Sativex | Sativex |
| BLINDED SATIVEX | Sativex |
| NABIXIMOLS (SATIVEX) OROMUCOSAL SPRAY | Sativex |
| SATIVEX (NABIXIMOLS) | Sativex |
| sativex | Sativex |
| SATIVEX - SPRAY PER MUCOSA ORALE | Sativex |
| DELTA?SATIVEX | Sativex |
| nabiximols | Sativex |
| Sativex Spray zur Anwendung in der Mundhoehle | Sativex |
| Sativex Spray | Sativex |
| Sativex Spray zur Anwendung in der Mundhohle | Sativex |
| FM2 OIL (MAGISTRAL PREPARATION) (NABIXIMOLS) | Sativex |
| Sativex Spray 38.5/41 mg | Sativex |
| Nabiximols | Sativex |
| MEVATYL | Sativex |
| sativex spray for application in the oral cavity | Sativex |
| sativex Spray zur Anwendung in der Mundhohle | Sativex |

**Epidiolex Terminology**

| **term** | **group** |
| --- | --- |
| Epidiolex | Epidiolex |
| EPIDIOLEX ODT | Epidiolex |
| EPIDIOLEX | Epidiolex |
| EPIDIOLEX CBD OIL | Epidiolex |
| EPIOLEX | Epidiolex |
| Epidolex | Epidiolex |
| Epidolex oral solution | Epidiolex |
| EPIDOLEX | Epidiolex |
| EPIDIOLEX (CBD) | Epidiolex |
| EPIDYOLEX (CANNABIDIOL) IN GERMAN CLINICAL PRACTICE | Epidiolex |
| EPIDIOLEX (RETAIL0 | Epidiolex |
| EPIDIOLEX (RETAIL) | Epidiolex |
| cannabidiol (Epidiolex) | Epidiolex |
| EPIDIOLEX SOL | Epidiolex |

**THC Terminology**

| **term** | **group** |
| --- | --- |
| TETRAHYDROCANNABINOL | THC |
| Tetrahydrocannabinol | THC |
| THC | THC |
| TETRAHYDROCANNABINOL (THC) | THC |
| CANNABIS (CANNABIS-TETRAHYDROCANNABINOL) | THC |
| 8-TETRAHYDROCANNABINOL | THC |
| DELTA-9-TETRAHYDROCANNABIONOL | THC |
| Delta-9-tetrahydrocannabinol | THC |
| DELTA-9-TETRAHYDROCANNABINOL | THC |
| 9-TETRAHYDROCANNABINOL | THC |
| UNSPECIFIED FORMULATIN OF TETRAHYDROCANNASINOL (THC) | THC |
| TETRAHYDROCANNABINOL (MARIJUANA,HASH) | THC |
| Marijuana/ THC/ hemp/Hash | THC |
| DELTA-9-CARBOXY-TETRAHYDROCANNABINOL | THC |
| 8-THC | THC |
| TETRANHYDROCANNABINOL | THC |
| tetrahydrocannabinol | THC |
| 11-NOR-9-TETRAHYDRO-9-CANNABINOLIC ACID | THC |
| DELTA(9)-TETRAHYDROCANNABINOL | THC |
| THC (PURE MARIJUANA IN PILL FORM) PRN | THC |
| TETRAHYDROCANNABINOL-THC | THC |
| THC (NOS) | THC |
| 8-Tetrahydrocannabinol | THC |
| 11-nor-9-carboxy-delta-9-tetrahydrocannabinol | THC |
| DELTA 9 THC | THC |
| TETRAHYDRO CANNABIDIAOL | THC |
| TETRAHYDRO CANNABIDIOL | THC |
| HYDROXY-THC | THC |
| DELTA-9 CARBOXY TETRAHYDROCANNABINOL | THC |
| 9-THC | THC |
| THC-COOH | THC |
| Carboxy- tetrahydrocannabinol | THC |
| 8?TETRAHYDROCANNABINOL | THC |
| Delta-9-Tetrahydrocannabinol | THC |
| thc | THC |
| Delta-9 THC | THC |
| Delta-9-carboxy THC | THC |
| THC TETRAHYDROCANNABINOL | THC |
| TETRAHYDROCANNABINOLS | THC |
| 11-NOR-9-CARBOXY-DELTA-9-TETRAHYDROCANNABINOL | THC |
| THC (9 TETRAHYDROCANNABINOL) | THC |
| THC (9 Tetrahydrocannabinol) | THC |
| low Indica thc | THC |
| .DELTA.8-TETRAHYDROCANNABINOL\HERBALS | THC |
| THC 3.4g Gummies | THC |
| THC Vaping | THC |
| DELTA 9-TETRAHYDROCANNABINOL | THC |
| 11-NOR-9-CARBOXY-?-9- TETRAHYDROCANNABINOL | THC |
| DELTA-9 CARBOXY THC | THC |
| 11-HYDROXY DELTA-9 THC | THC |
| DELTA-9 TETRAHYDROCANNABINOL | THC |
| 11-HYDROXY DELTA-9 TETRAHYDROCANNABINOL | THC |
| DELTA-9-CARBOXY-THC | THC |
| 11-HYDROXY-DELTA-9-TETRAHYDROCANNABINOL | THC |
| Delta-9-thc | THC |
| 11-NOR-9-CARBOXY-THC | THC |
| 9?TETRAHYDROCANNABINOL | THC |
| LEGAL LOQ THC CANNABIS | THC |
| [NO NAME] THC REFILL CARTRIDGE | THC |
| 11-Nor-9-carboxy-THC | THC |
| 11-COOH-THC (11-NOR-9-CARBOXY-.DELTA.9-TETRAHYDROCANNABINOL) | THC |
| TETRAHYDROCANNABINOL CARBOXYLIC ACID | THC |
| CANNABIS EKSTRACT (THC) | THC |
| Medical THC | THC |
| THC MARIJUANA | THC |
| SNOW SHARK 100MG THC CAPSULES | THC |
| 100MG THC | THC |
| TETRAHYDROCANNABINOL-CARBOXYLIC ACID | THC |
| Tetrahydrocannabin OL | THC |
| THC VAPE PEN | THC |
| VAPE DEVICE (THC OIL) | THC |
| THC OIL | THC |
| THC VAPING | THC |
| THC (Tetrahydrocannabinol) | THC |
| THC VAPING LIQUID | THC |
| PLATINUM AND DANK BRAND MARIJUANA CARTRIDGES (VAPE THC\NICOTINE) | THC |
| THE KIND PEN (USING GENERIC THC CARTRIDGE) | THC |
| DR. ZODIAKS (THC VAPING) | THC |
| HAVEY HITTERS (THC VAPING) | THC |
| THC VAPE | THC |
| HERBALS\TETRAHYDROCANNABINOL UNSPECIFIED | THC |
| Delta 9-tetrahydrocannabinol | THC |
| THC DROPS | THC |
| THC CARTRIDGE | THC |
| THC vape pen | THC |
| MARIJUANA HERB, THC OILS | THC |
| VAPE THC | THC |
| THC CARTRIDGES | THC |
| THC POWDER TO MIX WITH WATER FOR VAPING | THC |
| SUPREME CART THC DEVICE | THC |
| CARTNITE THC DEVICE | THC |
| SMOK NOVO THC VAPES | THC |
| VAPING THC OIL | THC |
| THC VAPE, DANK VAPES, EXOTIC CARTS, KING PEN, COOKIES, VAPURRR EXTRACT | THC |
| RYTHM SINGLE (DISPOSABLE THC VAPE) WWW.RYTHM.COM | THC |
| THC VAPE DANK STRAWBERRY | THC |
| THC DANK VAPES-HARDCORE OG | THC |
| Tetrahydrocannabinol Carboxylic Acid | THC |
| VAPE PEN WITH THC | THC |
| THC CONCENTRATES WAX | THC |
| DANK KING LOUIE [.DELTA.8-TETRAHYDROCANNABINOL\DEVICE\HERBALS] | THC |
| DANK STRAW-NANA FLAVORS [.DELTA.8-TETRAHYDROCANNABINOL\DEVICE\HERBALS] | THC |
| STIG THC-DANK VAPES, GLOW, CALIFORNIA CONFIDENTIAL | THC |
| TKO THC, FRUIT FLAVORING | THC |
| ^THC TOPPERS^ [.DELTA.8-TETRAHYDROCANNABINOL\DEVICE\HERBALS] | THC |
| ^THC TOPPERS-DANK, SUPREME, VSOP, COOKIE CART, ROVE, SMART CART AND MARIO CART [.DELTA.8-TETRAHYDROCANNABINOL\DEVICE\HERBALS] | THC |
| ^DABWOOD, ROVE, AND SUPREME G THC CARTRIDGES. | THC |
| DANK VAPES THC CARTRIDGES | THC |
| MEDICINAL THC 5 | THC |
| THC CARTRIDGE BRANDS: DANK VAPES. DOUGHBOY. GAS TANK | THC |
| thc - | THC |
| THC VAPE CARTRIDGE | THC |
| THC OIL VAPE PEN | THC |
| VALLEY LABS THC CARTRIDGES | THC |
| STRAIGHT FIRE VACUUM SEALED CARTS [THC] | THC |
| DABS, DAB WAX, DAB CARDS, WAX (THC VAPING) | THC |
| THC/NICOTINE VAPE CARTRIDGE | THC |
| CEREAL CARTS IN CEREAL FLAVORS [THC] | THC |
| DANK VAPES [THC] | THC |
| THC VAPING CARTRIDGES ARE UNFLAVORED: PINGS PENS, 1% TKO^ | THC |
| STRAINLY INTERNET SIGHT THC PLANTS (HEMP) | THC |
| VAPING THC | THC |
| DANK VAPES THC | THC |
| THC VAPES DANK | THC |
| THC [.DELTA.8-TETRAHYDROCANNABINOL] | THC |
| BRASS KNUCKLE [THC VAPE] | THC |
| THC VAPE JUICE | THC |
| DANK THC VAPE | THC |
| MEDICAL THC | THC |
| BIOTIN THC | THC |
| THC VAPE PENS | THC |
| DANK VAPES THC INFUSED E-LIQUID CARTS | THC |
| ROVE, DANK VAPES, AND CHRONIC BRANDS OF THC INFUSED E-LIQUIDS | THC |
| THC WAX MIXED WITH VAPEYOURWAX JUICE | THC |
| .DELTA.9-TETRAHYDROCANNABINOL\CANNABIDIOL\HERBALS | THC |
| .DELTA.8-TETRAHYDROCANNABINOL\DEVICE\HERBALS | THC |
| THC oil | THC |
| delta?9?tetrahydrocannabinol | THC |
| 9?TETRAHYDROCANNABIN OL | THC |
| 8?THC | THC |
| 9?THC | THC |
| Delta?9 Carboxy THC | THC |
| THC DROPS STENOCARE | THC |
| THC (9-CARBOXY THC) | THC |
| THC AND METABOLITES (TETRAHYDROCANNABINOL) | THC |
| THC (TETRAHYDROCANNABINOL) (THC-COOH) | THC |
| CANNTRUST THC DROPS | THC |
| 2600MG D8 SPECIAL EDITION EXTREME BROWNIE [.DELTA.8-TETRAHYDROCANNABINOL] | THC |
| THC?dominant cannabis | THC |
| THC?COOH | THC |
| TETRAHYDRO [TETRAHYDROCANNABINOL] | THC |
| Thc drops stenocare | THC |
| KOI DELTA 8 GUMMIES BLUE?RAZZ FLAVORED KOICBD.COM | THC |
| BEDROCAN (THC_PREDOMINANT) | THC |
| 11?NOR?9?CARBOXY?DELTA(9)?TETRAHYDROCANNABINOL | THC |
| CANNABURST THC GUMMY SOURS | THC |
| 11?nor?9?carboxy?delta?9?tetrahydrocannabinol | THC |
| Delta?9?tetrahydrocannabinol | THC |
| YOUR CURE CBD ? DELTA 8 VAPE TANK | THC |
| DELTA(9)?TETRAHYDROCANNABINOL | THC |
| THC PROSTAFLO [HERBALS] | THC |
| RAW GARDEN, THC VAPE PEN | THC |
| TETRAHYDROCANNABINOL CARBOXYLIC ACID (THC?COOH) | THC |
| Tetrahydrocannabinol (THC) | THC |
| UNKNOWN DELTA 8 THC GUMMY | THC |
| DELTA 8 THC | THC |
| THC BUTTERSCOTCH CHOCOLATE [DELTA 8 THC] | THC |
| 11-NOR-9-CARBOXY-.DELTA-9-TETRAHYDROCANNABINOL | THC |
| 11?NOR?9?CARBOXY?TETRAHYDROCANNABINOL | THC |
| 11?nor?9?carboxy?tetrahydrocannabinol | THC |
| DELTA?9 THC | THC |
| DELTA 8 THC GUMMIES | THC |
| DELTA 8 THC EDIBLE | THC |
| ZAR COSMIC CHOCOLATE CANDY BAR WITH PEANUT BUTTER [DELTA?8?THC] | THC |
| Delta 8 THC edible | THC |
| THC vape | THC |
| THC PROSTAFLO | THC |
| DELTA 8 THC CHOCOLATE | THC |
| 1000 MG THC DELTA 8 BROWNIE | THC |
| GALAXY TREATS MOON BABIES DELTA 8 THC GUMMIES 50 MG STARBERRY | THC |
| 8?Tetrahydrocannabinol | THC |
| THC CHOCOLATE BAR | THC |
| JAH GUMMIES WHAT?A?MELON [DELTA?8 THC] | THC |
| DELTA?8 CANNABIS | THC |
| BITES DELTA?8 THC PINK LEMONADE GUMMIS | THC |
| CANNAAID DELTA 8 OIL [.DELTA.8?TETRAHYDROCANNABINOL\HERBALS] | THC |
| .DELTA.8-TETRAHYDROCANNABINOL | THC |
| UNKNOWN THC GUMMY | THC |
| KUSH BURST THC GUMMIES PINEAPPLE PUNCH (DELTA 8) ? | THC |
| THC?0 | THC |
| THC EDIBLE | THC |
| THC W CANNABIS | THC |
| THC MARIJUANA | THC |
| 11-NOR-9-CARBOXY-.DELTA.9-TETRAHYDROCANNABINOL | THC |
| 11-HYDROXY-.DELTA.9-TETRAHYDROCANNABINOL | THC |
| .DELTA.9-TETRAHYDROCANNABINOL\HERBALS | THC |
| TETRAHYDROCANNABINOLS NOS | THC |
| HERBALS\TETRAHYDROCANNABINOL MIXED | THC |
| .DELTA.9-TETRAHYDROCANNABINOL ACETATE\HERBALS | THC |
| TETRAHYDROCANNABINOL UNSPECIFIED\HERBALS | THC |
| THC GUMMY | THC |
| TETRAHYDROCANNABINOLS NOS (TETRAHYDROCANNABINOLS NOS) | THC |
| THC Tincture | THC |
| DELTA 8 CBD | THC |
| THC W CANNIBIS | THC |
| Tetrahydrocannabinol nos | THC |
| Delta8THCDeath by GummyBear | THC |
| Tetrahydrocannabinol dab pen | THC |
| THC GUMMIES | THC |
| THC (CANNABIS) | THC |
| TETRAHDROCANNABINOL | THC |
| DELTA-9-CARBOXY THC | THC |
| TETRAHYDROCANNABIONAL | THC |
| TETRAHYDROCANNABINOL (TETRAHYDROCANNABINOL) | THC |
| TETRAHYDROCANNABINOL(TETRAHYDROCANNABINOL) | THC |
| TETRAHYDROCANNABINOL (TETRAHYDROCANNABINOL) CAPSULE, UNKNOWN | THC |
| TETRAHYDROCANNABINOL (TETRAHYDRACANNIBONOL) | THC |
| TETRAHYDROCANNABINOL (TETRATHYDROCANNABINOL) | THC |
| TETRAHYDROCANNABINOL (TETRAHYROCANNABINOL) | THC |
| TETRAHYDROCANABINOL (TETRAHYDROCANNABINOL) | THC |
| TETRAHYDROCOANNABINOL (TETRAHYDROCANNABINOL) | THC |
| TETRAHYDROCANNABINOL (TETRAHYDROCANNABINOL ) | THC |
| TETRAHYDROCANNABINOL (TETRAHYDROCANNABINOL) | THC |
| TETRAHYDROCANNABINOL (TETRAHYDROCANNABIMOL) | THC |
| TETRAHYDROCANNABINOL(TETRAHYDRCANNABINOL) | THC |
| TETRAHYDROCANNABINOL (TETRAHYDROCANNIBOL) | THC |
| TETRAHYDROCANNANBINOL(TETRAHYDROCANABINOL) | THC |
| TETRAHYDROCANNABINOL (TETAHYDROCANNABINOL) | THC |
| CARBOXY THC | THC |
| THC (TETRAHYDROCANNABINOL) (TETRAHYDROCANNABINOL) | THC |
| TETRAHYDROCANABINOL CARBOXY-ACID (TETRAHYDROCANNABINOL) (UNKNOWN) | THC |
| TETRAHYDROCANNABINOL (CON.) | THC |
| TETRAHYDROCANNABNIOL (TETRAHYDROCANNABINOL) | THC |
| THC-COOH (CANNABIS) | THC |
| THC (CANNABIS STAIVA) | THC |
| THC (CANNABIS SATIVA) | THC |
| DELTA-9-THC | THC |
| DELTA(9)-TETRAHYDROCANNABITOL | THC |
| DELTA (9) TETRA HYDRO CANNABINOL | THC |
| DELTA 9 TETRA HYDRO CANNABINOL | THC |
| THC (CANNABIS SATIVA) (INHALANT) | THC |
| DELTA-9-THC (TETRAHYDROCANNABINOL) | THC |
| THC (NO PREF. NAME) | THC |
| D-9-THC (NO PREF. NAME) | THC |

**THC/CBD Terminology**

| **term** | **group** |
| --- | --- |
| MARIJUANA SUPPLEMENTS (THC AND CBD) | THC/CBD |
| THC/cbd oil | THC/CBD |
| THC/CBD | THC/CBD |
| CANNABID CBD/THC | THC/CBD |
| TROKIE HYBRID CITRUS 0.98GR. 21.3MG THC, 19.9MG CBD, 20MG? | THC/CBD |
| TROKIE HYBRID CITRUS 0.98GR.21.3MG THC, 19.9MG CBD, 20MG? | THC/CBD |
| 5.6%CBD/3.7%THC DRONABINOL (VAPOR) | THC/CBD |
| CBD/THC oil | THC/CBD |
| CBD HEMP FLOWER (CBD\THC) | THC/CBD |
| BREEZ (CBD/THC) | THC/CBD |
| CBD;THC | THC/CBD |
| TETRAHYDROCANNABINOL 10MG;CANNABIDIOL 1MG | THC/CBD |
| TETRAHYDROCANNABINOL/CANNABIDIOL | THC/CBD |
| CANNABIDIOL, TETRAHYDROCANNABINOL | THC/CBD |
| CANNABIDIOL;DRONABINOL (TETRAHYDROCANNABINOL AND CANNABIDIOL) | THC/CBD |
| RELIEF VAPE PEN 1:9 CBD:THC | THC/CBD |
| SHIFT (CANNABIDIOL\.DELTA.8-TETRAHYDROCANNABINOL\DEVICE\HERBALS) | THC/CBD |
| CANNABIDIOL/DRONABINOL | THC/CBD |
| WANA SOUR GUMMIES 50:1 CBD/THC | THC/CBD |
| CANNTRUST 1:1 DROPS (CANNABIDOL/DRONABINOL) | THC/CBD |
| THC/CBD CARTRIDGE | THC/CBD |
| CBD/THC VAPING OIL | THC/CBD |
| THC/CBD OIL | THC/CBD |
| ORGANIC SMART CARTS (CBD\THC) | THC/CBD |
| LEGENDARY CARTS [THC/CBD] | THC/CBD |
| THC OIL, CANNABIDIOL FLAVORED WITH FRUIT PUNCH | THC/CBD |
| STRAINLY INTERNET SIGHT THC POLLEN (CBD, THC) | THC/CBD |
| THC VAPES DANK/VENOM/COOKIES/SMART KART/CALI PLUD [CANNABIDIOL\.DELTA.8-TETRAHYDROCANNABINOL\DEVICE\HERBALS] | THC/CBD |
| CANNABIDIOL OG DRONABINOL | THC/CBD |
| CANNABIDIOL;DRONABINOL | THC/CBD |
| CANNIMED OIL (CBD\THC) | THC/CBD |
| CBD/THC | THC/CBD |
| CANNABIDIOL,TETRAHYDROCANNABINOL | THC/CBD |
| DIAMOND STIXX [CBD\THC VAPE] | THC/CBD |
| CBD/THC TINCTURE 5MG | THC/CBD |
| Cannabidiol/tetrahydrocannabinol | THC/CBD |
| Cannabidiol;Dronabinol | THC/CBD |
| WANA SOUR GUMMIES EXOTIC YUZU CBD/THC | THC/CBD |
| MEDICAL CANNABIS RED 500 MG DISTILLATE PREFILLED VAPORIZER (CBD/THC) | THC/CBD |
| RED DISTILLATE PREFILLED VAPORIZER (CBD/THC) | THC/CBD |
| RAZZ BERRY 100 MG [CBD\THC] | THC/CBD |
| DELTA?8 50 MG CBD GUMMY | THC/CBD |
| CANNABIS INFUSED GUMMIES PLUS?BALANCE (CBD\THC) | THC/CBD |
| CBD WITH THC | THC/CBD |
| 50/50 edibles CBD/THC | THC/CBD |
| CANNABIS (MINT CAKE) (CBD\THC) | THC/CBD |
| DELTA 8 CBD FLOWER | THC/CBD |
| .DELTA.8-TETRAHYDROCANNABINOL\CANNABIDIOL\HERBALS | THC/CBD |
| COCOA PEBBLEZ TREATS (CBD\DELTA 8?THC) | THC/CBD |
| 7 G HYBRID TKO BY TERP NATION 8 HEMP FLOWER DELTA?8 (CANNABIDIOL\.DELTA.8?TETRAHYDROCANNABINOL\HERBALS) | THC/CBD |
| NAXIVA?PANAXIR T25C25 [CANNABIDIOL/DRONABINOL] | THC/CBD |
| DELTA 8 CBD GUMMIES 1000MG | THC/CBD |
| CBD DELTA 8 GUMMIES | THC/CBD |
| DELTA9 [CANNABIDIOL\.DELTA.8?TETRAHYDROCANNABINOL\DEVICE\HERBALS] | THC/CBD |
| CANNABIDIOL\.DELTA.8-TETRAHYDROCANNABINOL\HERBALS | THC/CBD |
| CANNABIDIOL\.DELTA.8-TETRAHYDROCANNABINOL | THC/CBD |
| CBD AND THC | THC/CBD |
| CANNABIDIOL\.DELTA.8-TETRAHYDROCANNABINOL\DEVICE\HERBALS | THC/CBD |
| CBD/THC SUPPLEMENT | THC/CBD |
| Cannabidiol;Tetrahydrocannabinols nos | THC/CBD |
| THC edible and CBD | THC/CBD |
| CANNABIDIOL\HERBALS\TETRAHYDROCANNABINOL MIXED | THC/CBD |
| THC AND CBD | THC/CBD |
| CANNABIDIOL\HERBALS\TETRAHYDROCANNABINOL UNSPECIFIED | THC/CBD |
| THC CBD PATCH | THC/CBD |
| CANNABIDIOL;TETRAHYDROCANNABINOL NOS | THC/CBD |
| CANNABIDIOL;TETRAHYDROCANNABINOLS NOS | THC/CBD |
| CANNABIDIOL + TETRAHYDROCANNABINOL | THC/CBD |
| potassium tropical cbd/thc 20 MEQ twice daily | THC/CBD |
| TETRAHYDROCANNABINOL AND CANNABIDIOL | THC/CBD |

**CBD Terminology**

| **term** | **group** |
| --- | --- |
| CANNABIDIOL\HERBALS | CBD |
| CANNABIDIOL OIL | CBD |
| CBD | CBD |
| CANNABIDIOL | CBD |
| CBD DROPS 200MG, 30ML SOOTHEEN | CBD |
| CBD OIL | CBD |
| PALMETTO HARMONY (CBD OIL) | CBD |
| JUNGLE JUICE CBD OIL 1ML JUST CHILL PRODUCTS | CBD |
| CBD IN HEMP OIL 1800 MG, IN 60 ML ABLE DOC.COM OR PROHELAHADVISOR | CBD |
| Cannabidiol | CBD |
| VAPE OIL WITH CBD ALTERNATE VAPE 5 ML (25 MG OF CBD) | CBD |
| HAYLEIGHS HOPE CBD OIL | CBD |
| CANNABIDOL | CBD |
| CBD OIL/CANNABINOID | CBD |
| CBD FOOD SUPPLEMENTS | CBD |
| cannabidiol | CBD |
| CBD OILS | CBD |
| CBD OIL-PALMETTO HARMONY | CBD |
| CBD - OIL | CBD |
| CBD OIL (MEDICAL MARIJUANA) | CBD |
| Medical Cannabis CBD | CBD |
| Cannabidiol oil | CBD |
| CBD Oil | CBD |
| CBD HEMP OIL IN MCT | CBD |
| CANNABIDIOL DRUPPELS 100MG/ML | CBD |
| HEMP BASED CBD OIL | CBD |
| CBD oil (NOS) | CBD |
| PUFCBD REMEDY KIT (POWER) | CBD |
| CBD oil | CBD |
| CBD drops | CBD |
| DIAMOND CBD | CBD |
| ADVANCED CBD OIL WITH TERPENES (FROM HEMP) | CBD |
| PEACE + WELLNESS ELEVATE (CBD/HEMP OIL INFUSED) | CBD |
| INFUSED BODY BUDDERS; INGREDIENT: CBD | CBD |
| MIRACLE BUDDER: UNSCENTED INGREDIENT: CBD | CBD |
| KANNAWAY CBD OIL | CBD |
| 20% CBD | CBD |
| CBD hemp oil | CBD |
| CANNABIDIOL ORAL SOLUTION (300MG/ML) | CBD |
| CBD, BATCH# TRU4720, PRODUCT# TRU4808, HARVEST# TRU4720 | CBD |
| MEDICAL CANNABIS OIL VIOLET CBD | CBD |
| CBD ORAL DROPS | CBD |
| CBD TYPE OF CANNABIS | CBD |
| CBD oral drops | CBD |
| LAB BLENDS CBD PAIN RELIEVER (LIDOCAINE\MENTHOL) | CBD |
| CANNABIDIOL\CANNABIS SATIVA SEED OIL (INACTIVE INGREDIENTS) | CBD |
| CHILL GUMMIES DIAMOND CBD | CBD |
| COSMIC CLEANSE BODY BUDDER (CBD) | CBD |
| HERBALOGIX CBD ENHANCED BODY LOTION | CBD |
| CBD Oils | CBD |
| CBD Salve | CBD |
| CBD Hemp Oil | CBD |
| medical cbd | CBD |
| CBD HEMP OIL | CBD |
| CBD lotion | CBD |
| CHARLOTTE'S WEB (CBD) | CBD |
| cbd oil | CBD |
| RED DEVIL KRATOM WATER SOLUBLE CBD | CBD |
| CBD OIL CRYSTAL ISOLATE 1500MG (AND GUMMIES) | CBD |
| CBD OIL (CANNABIDIOL) | CBD |
| CBD OIL PLUS | CBD |
| CBD HEMP FLOWER | CBD |
| CANNABIDIOL (CBD oil) | CBD |
| CBD Vape and Balm | CBD |
| CBD CREAM | CBD |
| CBD CANDIES | CBD |
| HERBAL INSANITY'S CBD OIL FOR PAIN | CBD |
| KUSH MASCARA (CBD OIL) | CBD |
| CBD oil / Hemp Oil | CBD |
| cbd | CBD |
| CBD balm | CBD |
| CBD cream | CBD |
| FLEXALL420 (CANNABIDIOL) | CBD |
| CANNABIDIOL ORAL SOLUTION 300 MG/ML | CBD |
| BIO REMEDIES PUFCBD | CBD |
| PUREKANA PREMIUM CBD OIL DROPS | CBD |
| CBD TINCTURE | CBD |
| cbd 5mg | CBD |
| CREATING BETTER DAYS PEACH POPS TANGY ORANGE 50MG CBD INFUSED | CBD |
| MEDICAL MARIJUANA/CBD | CBD |
| CBD GUMMY BEAR | CBD |
| HEMPWORK 750 WITH PURE CBD OIL AND HERBAL DROPS PEPPERMINT FLAVOR 1OZ/ | CBD |
| KOICBD PRODUCTS | CBD |
| CBDROP FULL SPECTRUM OIL | CBD |
| CBD OIL TABLET | CBD |
| Cannabidiol Oil | CBD |
| CANNABIDIOL ORAL SOLUTION 300MG/ML | CBD |
| EVOLUTION CBD OILS | CBD |
| LAZARUS NATURALS CBD CAPSULES 2000MG CBD | CBD |
| JUST CBD CANNABIDIOL GUMMIES GUMMY BEARS 250MG | CBD |
| optimacbd cbd 4% | CBD |
| CANNABIDIOL (CBD) | CBD |
| CBD WELLNESS CENTER DROPPER 1500MG | CBD |
| CBD paste | CBD |
| CBD EDIBLE | CBD |
| HEMPTRANCE NATURAL CBD GUMMIES | CBD |
| CBD OIL INA CREAM | CBD |
| CBD GUMMIES | CBD |
| QUEEN BEE NATURALS , CBD QUEEN | CBD |
| BATH BOMB [CANNABIDIOL] | CBD |
| CBD EXTRACT | CBD |
| CBD OIL 750 MG | CBD |
| CBD CANNABIS OIL (CANNABIDIOL) | CBD |
| CBD OIL, | CBD |
| CBD +STRESS DEFY | CBD |
| CBD HEMP | CBD |
| CBD hemp extract | CBD |
| CBD OIL (HEMP) | CBD |
| ULTRA CBD EXTRACT | CBD |
| CBD LOTION | CBD |
| CBD GUMMIES FROM HEMP 300 MG | CBD |
| PURE ALOE VERA GEL HEADACHE + SORE MUSCLES RUB [CBD] | CBD |
| PAIN STICK [CBD\MENTHOL] | CBD |
| CBD OIL | CBD |
| ELIXIR / CBD OIL TREATMENT | CBD |
| HEMP CBD | CBD |
| CBD ESSENTIAL OIL OINTMENT | CBD |
| MIRACLE LEAF CBD - EXTRA STRENGTH, 1200 MG | CBD |
| COOKIE INFUSED WITH CBD | CBD |
| CHILL RD VELVET TINCTURE CBD 1500 MG | CBD |
| CBD Alive | CBD |
| TRUORGANICS HIGH CBD OIL TINCTURE 900MG CBD | CBD |
| CBD Oil 50 mg | CBD |
| RELAX FULL SPECTRUM CBD OIL | CBD |
| SUNMED FULL SPECTRUM TINCTURE CBD OIL 750 MG | CBD |
| CBD KINGS | CBD |
| CBD TOBACCO TO RELAX | CBD |
| NATIVE LIPOSOMAL CBD EXTRACT SPRAY | CBD |
| RESET BIOSCIENCE BALANCE 300MG 99%+ NANO LIPOSOMAL ORGANIC HEMP CBD | CBD |
| CV SCIENCE CBD OIL | CBD |
| CBD CAPSULES | CBD |
| CBD VAPE PEN | CBD |
| PURIFIED CBD (CANNABIDLOL) ORA SOLUTION | CBD |
| DIAMOND CBD FULL SPECTRUM CBD OIL (TICLLOSENAC) | CBD |
| CBD (cannabidiol) | CBD |
| CBD SALVE | CBD |
| CBD OIL: 7MG CBD/1ML | CBD |
| CBD capsule | CBD |
| CBD TABLETS | CBD |
| KOI CBD | CBD |
| NEW LEAF CBD OIL | CBD |
| NOVO 2 VAPE PEN (CBD) | CBD |
| CBD EXTRACT OIL | CBD |
| CBD OIL 5M/DAY | CBD |
| ORGANABUS SILVER ORGANIC CBD VAPOR LIQUID | CBD |
| EVE'S MAGIC. HEMP REMEDY CBD OIL 750MG | CBD |
| AMOS HEMPS [CBD OIL IN MCT OIL] | CBD |
| CBD 35MG | CBD |
| CBD 25 MG | CBD |
| PLUS CBD OIL HEMP DROPS PEPPERMINT EXTRA STRENGTH | CBD |
| CBD FLAXSEES COMBO | CBD |
| CBD ADREXOL | CBD |
| LEAF AND FLOWER CBD SHAMPOO | CBD |
| LEAF AND FLOWER CBD CONDITIONER | CBD |
| CBDISTILLERY 33MG CBD PER SERVING FULL SPECTRUM HEMP SUPPLEMENT | CBD |
| HEMPLUCID CBD OIL 1000 MG VAPING | CBD |
| HEEL BALM WITH CBD AND MENTHOL | CBD |
| CHOCOLATE CHIP COOKIE [CBD] | CBD |
| JOY ORGANICS CBD OIL 500MG | CBD |
| CBD Kings | CBD |
| CTFO (CHANGING THE FUTURE OUTCOME) 10XPURE CBD HEMP OIL | CBD |
| CANNABIDIOL DRUPPELS, 50 MG/ML (MILLIGRAM PER MILLILITER) | CBD |
| JUST CBD GUMMIES | CBD |
| FEALS [CBD] | CBD |
| CBD OIL HEMP-DERIVED CANNABIDIOL FULL SPECTRUM HEMP SUPPL | CBD |
| JOINT VIBRANCE + CBD | CBD |
| CANNAVALLEY [CBD] | CBD |
| HEMP CLASSIC CBD | CBD |
| CTFO 10X PURE CBD GOLD 1000 | CBD |
| CBD-olie | CBD |
| COLESVAM MEDICAL MARIJUANA (CBD) | CBD |
| VAPING CBD OIL | CBD |
| MAGIC LEAF STRAWBERRY 1000MG/3ML CBD VAPE CARTRIDGE | CBD |
| MAGIC LEAF OGKOSH 1000MG/3ML CBD VAPE CARTRIDGE | CBD |
| cbd drops | CBD |
| SKIN KUSHION BY MONAT BODY BUTTER (CBD) | CBD |
| RSO CBD 2:1 | CBD |
| SOUL 500 [CANNABIDIOL\HERBALS] | CBD |
| CBD KINGS THC FREE | CBD |
| CTFO 10X GOLD CBD OIL AND 10X PURE ULTIMATE MULTI-VITAMIN | CBD |
| VAPORIZER CBD/MARIJUANA | CBD |
| CBD VAPING | CBD |
| QUEEN CITY HEMP 500MG (CBD) | CBD |
| queen city hemp cbd | CBD |
| Cbd | CBD |
| cbd hemp oil | CBD |
| CBD/CBD OIL VAPE | CBD |
| CHARLOTTE'S WEB STANLEY BROTHERS 17MG OLIVE OIL 30ML (CBD) | CBD |
| HEMPWORX CBD OIL | CBD |
| PREMIUM CBD DROPS | CBD |
| ORIGINAL FORMULA HEMP EXTRACT OIL MINT CHOCOLATE FLAVOR CHARLOTTES WEB [CBD] | CBD |
| ORIGINAL FORMULA HEMP EXTRACT OLIVE OIL FLAVOR CHARLOTTES WEB [CBD] | CBD |
| cbd kings | CBD |
| CBD BALM - EXTRA STRENGTH | CBD |
| NULEAF NATURALS FULL SPECTRUM CBD OIL | CBD |
| CBD DROPS (ONYX + ROSE BROAD SPECTRUM) | CBD |
| cannabidiol oil | CBD |
| CBD GUMMY | CBD |
| TURMERIC + CBD | CBD |
| RESCUE BLEND RAW ORGANIC HONEY WITH CBD 250 MG | CBD |
| FULL SPECTRUM CBD OIL 850MG | CBD |
| CBD Extract | CBD |
| Rescue Blend Raw Organic Honey with CBD 250 mg | CBD |
| ULTIMATE CBD VAPE ADDITIVE (CBD OIL) | CBD |
| CBD ointment | CBD |
| CBD CREAM (BRAND/FORMULATION UNKNOWN) | CBD |
| CBDFX HEMP GUMMIES | CBD |
| CBD DROPS | CBD |
| CANNABIDIOL. | CBD |
| CALM REST AND RELAX HEMP EXTRACT FORMULA (CBD) | CBD |
| ARTISANAL CANNABIDIOL | CBD |
| OPTIFORM CANNABIDIOL (CBD) | CBD |
| LIBERTY CBD TINCTURE, JACKSON'S COURAGE | CBD |
| NULEAF NATURALS CBD | CBD |
| WBRX METERED DOSE INHALER [CANNABIDIOL\DEVICE\HERBALS] | CBD |
| CBD Oil (NON-ABBVIE) | CBD |
| SUNMED VEGAN GUMMY BEARS WITH 5MG CBD | CBD |
| SOUR WORMS 10 PIECE [CBD] | CBD |
| Cbd kings | CBD |
| T?Relief CBD+13 Sublingual | CBD |
| CBD GUMMY 3MG | CBD |
| CYPRESS HEMP CBD OMEGAS | CBD |
| CANN I BE SO EXTRA TROP THE BEAT 1,000 MG FULL SPECTRUM CBD OIL | CBD |
| LIFE STREAMS CBD GUMMY | CBD |
| CRISP CBD | CBD |
| PRO RESTORE CBD+ | CBD |
| CBD INTENSIVE CREAM | CBD |
| CANNABIDIOL (CBD) OIL | CBD |
| HAWAIIAN HAZE CBD 18.9% | CBD |
| EXTRA STRENGTH TABLETS CBD GEL | CBD |
| Cbd oil | CBD |
| INV cannabidiol 300mg/ml oral solution | CBD |
| CBD OINTMENT | CBD |
| CBD products | CBD |
| CBD Kings with Lidocaine | CBD |
| CBD (NO THC) | CBD |
| CBD KINGS EX | CBD |
| CANNABIDIOL CREAM | CBD |
| CBD salve | CBD |
| FULL SPECTRUM CBD CAPSULES | CBD |
| LEVEN CANNABIDIOL 1500MG CBD SERUM | CBD |
| Palmetto Harmony CBD Oil | CBD |
| Jade CBD Oil | CBD |
| Mary's CBD Oil | CBD |
| Charlottes Web CBD Oil | CBD |
| HI STEVIE CBD TINCTURE | CBD |
| Liberty CBD Tincture, Jackson's Courage | CBD |
| ADVEN (CANNABIDIOL) | CBD |
| CBD (Cannabidiol) | CBD |
| SWAG HEMP INFUSED NATURAL CBD GUMMIES | CBD |
| CBD Oil (Cannabidiol) | CBD |
| SWANSON ULTRA? CBD + SLEEP SUPPORT FULL SPECTRUM | CBD |
| CBD KINGS DIS W/LIDO | CBD |
| CBD stuff | CBD |
| ^HARRELSON'S OWN^ CBD OIL | CBD |
| Charlotte's Web CBD | CBD |
| CBD (CANNABIDIOL) | CBD |
| CBD-KINGS WITH LIDOCAINE | CBD |
| CBD (CANNABIDIOL) OIL (DIETARY SUPPLEMENT) | CBD |
| CBD SUPPLEMENTS | CBD |
| CBD COMPLEX | CBD |
| CBD Liquid | CBD |
| NOT POT ORIGINAL CBD GUMMIES | CBD |
| CANNABLISS BALM | CBD |
| CBD GEL CAP | CBD |
| CBD PRODUCT | CBD |
| CBD INFUSED VAGINAL TIGHTENING OIL | CBD |
| CBD W/MELATONIN | CBD |
| CANNABIOL | CBD |
| CBD EDIBLE CINNAMON COOKIE BAR 475MG | CBD |
| Cannabiol | CBD |
| CBD DROPS LUCID BLOOD ORANGE 1000MG CBD HEMP EXTRACT | CBD |
| MR. CBD OXYGENATED HEMP OIL | CBD |
| CHARLOTTE'S WEB ORIGINAL FORMULA CBD | CBD |
| cannabidiol (CBD) gummy candies | CBD |
| vaporized CBD | CBD |
| HEMP cbd | CBD |
| CBD WAX PRODUCT (CBD) | CBD |
| Mary's CBD | CBD |
| Palmetto Harmony CBD | CBD |
| Jade Nectar CBD | CBD |
| LEAF THERAPEUTICS CBD SLEEP BLEND (DIETARY SUPPLEMENT\HEMP) | CBD |
| CBD 25 MG | CBD |
| RADICAL RELEIF ANTI?INFLAMMATORY CBD PILLS | CBD |
| TILRAY CBD | CBD |
| CBD + MAGNESIUM | CBD |
| CBD TABLET | CBD |
| CANNABIDIOL EXTRACT | CBD |
| CBD product | CBD |
| CBD AND MELATONIN | CBD |
| CANNABIDIOL Powder | CBD |
| CBD Kings Dis W/Lido | CBD |
| UNKNOWN CBD GUMMY | CBD |
| CBD COOKIES | CBD |
| Melatonin/CBD | CBD |
| CBD Cream | CBD |
| Haleigh^s Hope CBD Oil | CBD |
| VAPORIZED CANNABIDIOL (CBD) OIL | CBD |
| CBD Oil cream | CBD |
| Medical marijuana and CBD | CBD |
| Hemp organic CBD | CBD |
| CBD CANNABIS | CBD |
| shikai CBD body lotion | CBD |
| CBD kings | CBD |
| CBD CAPSULE | CBD |
| CANNABIDIOL\CANNABIS SATIVA SEED OIL | CBD |
| CBD oil 1cc daily | CBD |
| CBD-KINGS | CBD |
| CBD whole plant | CBD |
| CBD Oil Kings | CBD |
| CBD PAIN RELIEF CREAM | CBD |
| Low-dose CBD oil | CBD |
| CBD OEL | CBD |
| canabediol | CBD |
| CBD gummies, 25 mg, 2, bid | CBD |
| CBD OILSUPLLIMENT | CBD |
| Melatonine with CBD | CBD |
| cbd oil tincture | CBD |
| CBD for sleep | CBD |
| CANABIDIOL | CBD |
| CANNABIDOLOL | CBD |
| CBD (NON-ABBVIE) | CBD |
| CBD softgel | CBD |
| cannabidoil | CBD |
| CBD rub | CBD |
| CBD tincture | CBD |
| CBD Kings THC Free | CBD |
| Cbdfx hemp gummie | CBD |
| CBD Balm | CBD |
| Cbdfx hemp gummies | CBD |
| Cannabidiol glostrup | CBD |
| CBD gummies(for pain) | CBD |
| OTHER THERAPEUTIC PRODUCTS (CBD king dis w/lido) | CBD |
| CBD gel caps | CBD |
| full-spectrum CBD | CBD |
| Cbd hemp | CBD |
| CBD PATCHES | CBD |
| CBD PATCH | CBD |
| CBD Cannabidiol | CBD |

**Cannabis Terminology**

| **term** | **group** |
| --- | --- |
| CANNABIS | Cannabis |
| CANNABIS SATIVA SUBSP. INDICA TOP | Cannabis |
| MEDICAL CANNABIS | Cannabis |
| MEDICAL MARIJUANA (MARIJUANA) | Cannabis |
| CANNABIS SATIVA | Cannabis |
| Cannabis | Cannabis |
| MARIJUANA | Cannabis |
| Medical Marijuana | Cannabis |
| Cannabis sativa | Cannabis |
| CANNABIS SATIVA (CANNABIS) (UNKNOWN) | Cannabis |
| MEDICAL MARIJUANA | Cannabis |
| CANNABIS (CANNABIS) | Cannabis |
| Marijuana | Cannabis |
| MARIJUANA (CANNABIS SATIVA) (CANNABIS SATIVA) | Cannabis |
| CANNABIS SATIVA OIL | Cannabis |
| MEDICINAL MARIJUANA | Cannabis |
| MARIJUANA (CANNABIS SATIVA)(CANNABIS SATIVA) | Cannabis |
| MARIJUANA (CANNABIS SATIVA ) | Cannabis |
| MARIJUANA (CANNABIS) (PILL) | Cannabis |
| MARIHUANA | Cannabis |
| UNSPECIFIED ^CANNABINOIDS^ | Cannabis |
| Oil with cannabis | Cannabis |
| MARIJUANA (MARIJUANA) | Cannabis |
| CANNABINOIDS | Cannabis |
| Cannabis Sativa | Cannabis |
| OIL MARIJUANA | Cannabis |
| CANNABIS\CANNABIS SATIVA L | Cannabis |
| WHITE RECLUSE MARIJUANA FLOWERS | Cannabis |
| Medical marijuana | Cannabis |
| MARIJUANA (CANNIBIS SATIVA) | Cannabis |
| CANNABIS (CANNABIS SATIVA) UNKNOWN | Cannabis |
| CANNABIS SATIVA SUBSP. SATIVA FLOWERING TOP | Cannabis |
| CANNABIS RESIN | Cannabis |
| Cannabinoids | Cannabis |
| marijuana | Cannabis |
| Marijuana Kush | Cannabis |
| MARIJUANA FOR MEDICAL USE | Cannabis |
| ILLEGAL MARIJUANA | Cannabis |
| Medicinal Marijuana | Cannabis |
| Cannabis oil | Cannabis |
| CANNABIS OILS | Cannabis |
| CANNABIS SATIVA FLOWERING TOP | Cannabis |
| MEDICAL MARIJUANA (PRESCRIPTION) | Cannabis |
| CANNABINOID | Cannabis |
| cannabis | Cannabis |
| MARIJUANA WAX | Cannabis |
| CANNABIS AND RESIN | Cannabis |
| MEDICINAL CANNABIS | Cannabis |
| medical marijuana | Cannabis |
| CANNABIS SATIVA FLOWER | Cannabis |
| CANNABINIODS | Cannabis |
| Vaporized Marijuana | Cannabis |
| CANNABIS SUBOXONE | Cannabis |
| CANNABINOIDS OIL | Cannabis |
| Cannabis inhalation | Cannabis |
| RECREATIONAL MARIJUANA | Cannabis |
| Medical Cannabis | Cannabis |
| CANNABIS DROPS | Cannabis |
| CANNABIS INDICA | Cannabis |
| Cannabis Oil | Cannabis |
| CANNABIS TEA | Cannabis |
| CANNABIS OIL | Cannabis |
| FULL CANNABIS OIL | Cannabis |
| LEGAL MARIJUANA | Cannabis |
| CANNABIS HERBAL EXPEC | Cannabis |
| MEDICAL MARIJUANNA | Cannabis |
| BEDROCAN MEDICINAL CANNABIS | Cannabis |
| CANNABIS (BEDROBINOL) | Cannabis |
| MEDICINAL RECREAT CANABIS HEMP F | Cannabis |
| CANNABIS SATIVA FRUIT | Cannabis |
| CANNABIS (CANNABIS SATIVA) | Cannabis |
| cannabis bedica olie | Cannabis |
| TART CHERRY JUICE AND NATURAL REMEDIES FOR PAIN SUCH AS CANNABIS | Cannabis |
| vaporized medicinal marijuana | Cannabis |
| cannabis oil | Cannabis |
| MARIJUANA OILS | Cannabis |
| cannabis bedica | Cannabis |
| CANNABIS COOKIE | Cannabis |
| INDIGO MARIJUANA | Cannabis |
| MARIJUANNA | Cannabis |
| Medical cannabis | Cannabis |
| HOMEMADE CANNABIS OIL | Cannabis |
| MARIJUANA TEA | Cannabis |
| 61% Cannabis oil | Cannabis |
| CANABIS LOTION | Cannabis |
| medicinal marijuana | Cannabis |
| 100% HEMP EXTRA VIRGIN NO THC | Cannabis |
| ACETAMINOPHEN/CODINE MEDICAL MARIJUANA | Cannabis |
| MEDICAL CANNABIS PATIENT CERTIFIED | Cannabis |
| CANNABIS PREPARATION | Cannabis |
| Cannabis indica | Cannabis |
| MEDICAL MARIJUANA OIL | Cannabis |
| MEDICAL GRADE CANNABIS | Cannabis |
| CANNABIS/CANNABIS SATIVA | Cannabis |
| CANNABANOIDS | Cannabis |
| CANNABIS INFUSED DARK CHOCOLATE | Cannabis |
| Marihuana | Cannabis |
| MARIJUANA EXTRACT | Cannabis |
| Cannabis sativa oil | Cannabis |
| medical cannabis | Cannabis |
| CANNABIS EXTRACT OIL | Cannabis |
| CANNABIS inflorescences | Cannabis |
| CANNABIS ET RESINE | Cannabis |
| CANNABIS, INDICA | Cannabis |
| BLACK MAMBA (CANNABIS SATIVA) | Cannabis |
| CANNABIS AND OIL | Cannabis |
| MEDICINAL CANNABIS OIL | Cannabis |
| Medicinal Cannabis | Cannabis |
| CANNABIS SATIVA SEED OIL\HERBALS | Cannabis |
| MEDICAL CANNABIS TINCTURE | Cannabis |
| MEDICAL CANABIS | Cannabis |
| CANNABIS and RESINE | Cannabis |
| Cannabis (cannabis sativa) | Cannabis |
| RICK SIMPSON CANNABIS OIL | Cannabis |
| medical Cannabis oil | Cannabis |
| MEDICAL MARIJUANA (RSO) | Cannabis |
| CANNABIS OIL, IN THE FORM OF A CHEWY GUMMY PRODUCT | Cannabis |
| medical marijuane | Cannabis |
| CANNABIS SATIVA SEED\HERBALS | Cannabis |
| Edible marijuana | Cannabis |
| CANNABINOID PRODUCT NOS | Cannabis |
| MARIJUANA OIL | Cannabis |
| MED MARIJUANA | Cannabis |
| Canabinoid Oil | Cannabis |
| cannabinoids | Cannabis |
| TOPICAL CANNABIS | Cannabis |
| MARIJUANA LIQUID | Cannabis |
| CANNABIS SATIVA OIL;COLECALCIFEROL | Cannabis |
| Canabis | Cannabis |
| Unspecified Medical Marijuana | Cannabis |
| Medical Marijuana Tincture Oils | Cannabis |
| CANNABIS BUD/FLOWER | Cannabis |
| TWEED CANNABIS OIL | Cannabis |
| ^Marijuana cream^ | Cannabis |
| medical cannabis | Cannabis |
| CANNABIS INDICA SMOKE | Cannabis |
| TRULIEVE [MEDICAL CANNABIS] | Cannabis |
| CANNABINOIDS NOS | Cannabis |
| VAPE PEN (MARIJUANA) | Cannabis |
| CANNABIS products | Cannabis |
| MARIJUANA FLOWER | Cannabis |
| CANNABIS SATIVA EXTRACT | Cannabis |
| CANNABIS OIL EXTRACT | Cannabis |
| PAX 3 VAPORIZER ^NOTHING WAS OBTAINED, BUT DRIED MARIJUANA WAS USED IN THE PAX 3 DEVICE | Cannabis |
| MARIJUANA OIL VAPING | Cannabis |
| DISPENSARY MARIJUANA PLANT AND WAX CARTRIDGES. | Cannabis |
| CANNABIS CARTRIDGE | Cannabis |
| CANNABIS VAPE CARTRIDGE WITH PHYTOL | Cannabis |
| VAPING MARIJUANA | Cannabis |
| MARIJUANA HERB DEVICE USED: LOKEE BRAND VAPE^ | Cannabis |
| MARIJUANA VAPING LIQUID | Cannabis |
| CANNABIS VAPING | Cannabis |
| MARIJUANA VAPE | Cannabis |
| MARIJUANA VAPING PRODUCT | Cannabis |
| HASH OIL [CANNABIS OIL VAPING] | Cannabis |
| WAX, MARIJUANA | Cannabis |
| CLAW VAPE PEN ^THE POD^ ORANGE KUSH OIL STIX (CANNABIS OIL) | Cannabis |
| ABSOLUTE XTRACTS ^GSC^ CANNABIS OIL VAPE CARTRIDGE | Cannabis |
| Marijuana tincture | Cannabis |
| MARIJUANNA E-CIGARETTES | Cannabis |
| ARMOUR THYROID 60MG MEDICAL MARIJUANA PRN | Cannabis |
| edible marijuana | Cannabis |
| MEDICAL MARIJUANA CARD HOLDER | Cannabis |
| Apothecanna with cannabis | Cannabis |
| Cannabies | Cannabis |
| YELLOW CANNABIS SOFTGELS (HYBRID) BY SPECTRUM CANNABIS | Cannabis |
| HYBRID CANNABIS | Cannabis |
| MARIHUANA MEDICINE | Cannabis |
| MARIJUANA EDIBLES | Cannabis |
| 60MG, PLANT?BASED CANNABINOIDS PER 1ML HEMP EXTRACT MINT CHOCOLATE FLA | Cannabis |
| Marijuana Gummies | Cannabis |
| cannabis sativa | Cannabis |
| BLUE CANNABIS SOFTGELS (HYBRID) BY SPECTRUM CANNABIS | Cannabis |
| Marijuana drops | Cannabis |
| MEDICAL MARIJUANA OIL VAPOR | Cannabis |
| Medical Marijuana Tinchur | Cannabis |
| CANNAB (MARIJUANA DROPS) | Cannabis |
| Medicinal cannabinoid extract | Cannabis |
| Cannabis spray | Cannabis |
| CANNABINOID OIL | Cannabis |
| CANNABIS OIL AND CREAM | Cannabis |
| Smoked cannabis | Cannabis |
| Vapes Cannabis | Cannabis |
| Smokes Marijuana | Cannabis |
| Smokes marijuana | Cannabis |
| smokes marijuana | Cannabis |
| CANNAB | Cannabis |
| CANNABIS SATIVA SEED OIL | Cannabis |
| MARIJUANA, N.O.S | Cannabis |
| MARIJUANA, N.O.S. (CANNABIS SATIVA) | Cannabis |
| CANNABIS FLOS BEDIOL (CANNABIS SATIVA FLOWER) | Cannabis |
| Marijuana oil | Cannabis |
| CANNABIS ? HEALTH CANADA | Cannabis |
| CANNABIS SATIVA E SEMINIBUS | Cannabis |
| Therpeutic cannabis | Cannabis |
| medical marijuanas | Cannabis |
| Marijuana Oil | Cannabis |
| MARIJUNAN | Cannabis |
| Smokes Cannabis | Cannabis |
| CANNABIS OEL | Cannabis |
| CANNABIS MEDICAL | Cannabis |
| CANNABIS FLOS | Cannabis |
| CANNABIS SATIVA SUBSP. INDICA | Cannabis |
| CANNABINOIDS(CANNABINOIDS) | Cannabis |
| CANNABIS SATIVA VAR. INDICA | Cannabis |
| Cannabis oel | Cannabis |
| CANNABIS OINTMENT | Cannabis |
| Marijuana edible | Cannabis |
| MEDICINAL CANNABIS CAPSULES | Cannabis |
| Recreational marijuana | Cannabis |
| Cannabis- smokes | Cannabis |
| cannabis cake | Cannabis |
| cannabis- smokes | Cannabis |
| Medical Indica Marijuana | Cannabis |
| Medical Sativa Marijuana | Cannabis |
| 20164 (GLOBALC3Sep21): Medical Marijuana | Cannabis |
| CANNABIS VAPE OIL | Cannabis |
| Cannabinoid | Cannabis |
| MARIJUANA TINCTURE | Cannabis |
| CANNABIS SATIVA LEAF | Cannabis |
| MARIJUANA GUMMIES | Cannabis |
| medicinal cannabis | Cannabis |
| MEDICAL MARIJUANAS | Cannabis |
| MEDICAL MARIJUANA (RICK SIMPSON OIL) | Cannabis |
| MARIJUANA VAPOR | Cannabis |
| CANNABIS SATIVA FLOWER;CANNABIS SATIVA SEED OIL | Cannabis |
| CANNABINOLIC ACID | Cannabis |
| Medical-Marijuana | Cannabis |
| CANABIS | Cannabis |
| Canabis oil | Cannabis |
| Cannabis gummies | Cannabis |
| MARIJUANA (OPIATE UNSPEC.) | Cannabis |
| MARIJUANA USE | Cannabis |
| MARIJUANA (CANNABIS) | Cannabis |
| MARIJUANA UNKNOWN (CANNABIS) | Cannabis |
| MARIJUANA(OPIAT) | Cannabis |
| MARIJUANA(CANNABIS) | Cannabis |
| CANNABIS (FORMULATION UNKNOWN) (CANNABIS) | Cannabis |
| CANNABIS(CANNABIS) (UNK) | Cannabis |
| MARIJUANA (CANNABIS, ) | Cannabis |
| CANNABIOIDS | Cannabis |
| MARIJUANA METABOLITES | Cannabis |
| MARIJUANA (CANNABIS | Cannabis |
| CANNABINODIS | Cannabis |
| CANNABNOIDS (CANNABIS) | Cannabis |
| CANNABOIDS | Cannabis |
| CANNABIS SATIVA(CANNABIS) | Cannabis |
| CANNABIS(CANNABIS) | Cannabis |
| MARIJUANA(CANNABIS) UNKNOWN | Cannabis |
| MARIJUANA ( CANNABIS) | Cannabis |
| CANNABIS (CANNABIS) | Cannabis |
| MARIJUNA | Cannabis |
| MARIJUANA (CANNABIS) UNKNOWN | Cannabis |
| CANNABIS (CANNABIS, EXTENDED RELEASE) | Cannabis |
| CANNABINOIDS (CANNABIS) | Cannabis |
| CANNABINOIDS () | Cannabis |
| CANNABIS (CAQNNABIS) | Cannabis |
| CANNABIS (CANNABIS, ) | Cannabis |
| CANNABIS (CANNABIS, , 0) | Cannabis |
| MARKUUANA (CANNABIS) | Cannabis |
| MARIJUANA (CANNIBIS) | Cannabis |
| CANNABINOIDS() | Cannabis |
| MARIJUANA (CANNIBAS) | Cannabis |
| MARIJUANA (CANNABIS)UNKNOWN | Cannabis |
| CANABANOIDS (CANNABIS) | Cannabis |
| MARIJUANA (CANNABIS) | Cannabis |
| MARUANA (CANNABIS) | Cannabis |
| CANNABIS SMOKING | Cannabis |
| MARIJUANA(MARIJUANA) | Cannabis |
| MARIJUANA (CANNABIS SATIVA) | Cannabis |
| CANNABIS (FORMUATION UNKNOWN) (CANNABIS) | Cannabis |
| MARIJUAN (CANNABIS) | Cannabis |
| MARIJUANA (CANNABIS,) | Cannabis |
| MARIJUANA (CANNABIS SATIVA) | Cannabis |
| CANNABIS (CANNIABIS) | Cannabis |
| MARIJUANA (CANNABIS | Cannabis |
| MARIJUANA (MARIJUANA) | Cannabis |
| CANNABIS(CANNABIS, CANNABIS SATIVA) | Cannabis |
| CANNABIS (CANNABIS, 0) | Cannabis |
| CANNABIS (CANNABIS0 | Cannabis |
| CANABIS (CANNABIS) | Cannabis |
| MARIJUNA (CANNABIS) | Cannabis |
| MARIJUANA (CANNABIS0 | Cannabis |
| MEDICINAL MARIHUANA | Cannabis |
| CANNABIS (CANNABIS) | Cannabis |
| CANNABIS SATIVA(CANNABIS, CANNABIS SATIVA) | Cannabis |
| MARIJUANA (CANNABIS, CANNABIS SATIVA) | Cannabis |
| CANNABIS (CANNABIS, CANNABIS SATIVA) | Cannabis |
| 2 HITS OF MARIJUANA | Cannabis |
| MARIJUANA (CANNABIS) | Cannabis |
| MARIJUANA (MEDICAL) | Cannabis |
| MEDICINAL MARIJUANA (CANNABIS SATIVA) | Cannabis |
| CANNABIS (CANNABIS SATIVA) (CON.) | Cannabis |
| LEGALIZED MARIJUANA | Cannabis |
| CANNABIS (HASHISH - CANNABIS) | Cannabis |
| MEDICAL MARIJUANA (CANNABIS SATIVA) | Cannabis |
| MARIJUANA/SYNTHETIC PILL FORM OF MARIJUANA (CANNABIS SATIVA) | Cannabis |
| CANNABIS (TABLETS) | Cannabis |
| MARIJUANA (CANNABIS, CANNABIS SATIVA) | Cannabis |
| MARIJUA | Cannabis |
| MARIJUANA (CANNABIS SATIVA) UNKNOWN | Cannabis |
| MARIJUANA TBD HERB MEDICAL CENTER, 12509 OXNARD ST. N.HOLLYW | Cannabis |
| MARIJUANA (MARIJUANA) UNKNOWN | Cannabis |
| CANNABIS PREPARATION (CANNABIS PREPARATION) | Cannabis |
| MARIJUANA (NO PREF. NAME) | Cannabis |
| CANNABIS TRANSPLACENTAL | Cannabis |
| DRUG - MARIJUANA | Cannabis |
| CANNABIS SATIVA (Hemp hearts) | Cannabis |
| CANNABIS LOTIONS | Cannabis |
| CANNABIS SATIVA (CANNABIS SATIVA) | Cannabis |
| CANNABIS (NO PREF. NAME) | Cannabis |
| CANNABIS (NONE) | Cannabis |
| CANNABIS (CANNABIS SATIVA | Cannabis |
| CANNABINOIDS (NO PREF. NAME) | Cannabis |
| CANNABIS (CANNIBIS SATIVA) | Cannabis |
| THC FREE | Cannabis |
| THC Free | Cannabis |
| THC FREE LIQ 20MG/ML | Cannabis |
| 100% HEMP EXTRA VIRGIN NO THC | Cannabis |

**Online Appendix 2** The top 15 disproportionality analysis estimates for Epidiolex (top panel) and CBD (bottom panel) at the preferred term level, ranked by IC_025_ (FAERS Q2 2018 to Q1 2023)

| **PT** | **Cases** | **ROR (95% CI)** | **PRR (95% CI)** | **IC (95% CrI)** |
| --- | --- | --- | --- | --- |
| ***Epidiolex*** |  |  |  |  |
| Seizure cluster | 86 | 219.04 (170.54 to 281.34) | 217.68 (169.66 to 279.27) | 6.36 (6.05 to 6.68) |
| Change in seizure presentation | 60 | 204.31 (151.86 to 274.88) | 203.43 (151.35 to 273.43) | 6.07 (5.68 to 6.45) |
| Atonic seizures | 59 | 191.44 (142.30 to 257.55) | 190.62 (141.83 to 256.21) | 6.02 (5.64 to 6.41) |
| Seizure | 4169 | 68.95 (66.41 to 71.58) | 48.34 (47.06 to 49.65) | 5.47 (5.43 to 5.51) |
| Weight abnormal | 126 | 72.00 (59.76 to 86.75) | 71.35 (59.30 to 85.83) | 5.67 (5.40 to 5.93) |
| Product supply issue | 298 | 46.99 (41.70 to 52.95) | 45.99 (40.91 to 51.7) | 5.31 (5.14 to 5.48) |
| Anticonvulsant drug level increased | 48 | 109.77 (80.49 to 149.71) | 109.39 (80.29 to 149.05) | 5.56 (5.14 to 5.99) |
| Emergency care | 124 | 55.51 (46.11 to 66.81) | 55.01 (45.77 to 66.12) | 5.39 (5.12 to 5.65) |
| Product administration interrupted | 205 | 36.55 (31.70 to 42.14) | 36.02 (31.30 to 41.45) | 4.97 (4.77 to 5.18) |
| Sudden unexplained death in epilepsy | 20 | 72.37 (45.39 to 115.39) | 72.27 (45.35 to 115.16) | 4.66 (3.95 to 5.36) |
| Drooling | 84 | 21.28 (17.18 to 26.60) | 21.25 (17.10 to 26.41) | 4.20 (3.86 to 4.54) |
| Prescribed overdose | 196 | 16.64 (14.42 to 19.20) | 16.42 (14.26 to 18.91) | 3.94 (3.72 to 4.16) |
| Product distribution issue | 84 | 17.24 (13.87 to 21.44) | 17.14 (13.81 to 21.29) | 3.93 (3.59 to 4.27) |
| Therapy responder | 13 | 137.44 (74.83 to 252.45) | 137.31 (74.79 to 252.09) | 4.45 (3.56 to 5.33) |
| Generalized tonic-clonic seizure | 183 | 14.77 (12.74 to 17.12) | 14.59 (12.61 to 16.88) | 3.78 (3.55 to 4.01) |
| ***CBD*** |  |  |  |  |
| Multiple-drug resistance | 65 | 94.66 (73.66 to 121.63) | 93.04 (72.71 to 119.06) | 5.73 (5.35 to 6.12) |
| Blood pressure diastolic decreased | 64 | 39.24 (30.58 to 50.35) | 38.59 (30.20 to 49.32) | 4.88 (4.49 to 5.27) |
| Device related thrombosis | 24 | 96.10 (63.73 to 144.91) | 95.49 (63.49 to 143.64) | 5.00 (4.36 to 5.65) |
| Malignant cranial nerve neoplasm | 17 | 8561.53 (2879.45 to 25456.18) | 8522.92 (2869.16 to 25317.61) | 5.10 (4.33 to 5.87) |
| Retro-orbital neoplasm | 17 | 2140.38 (1080.62 to 4239.44) | 2130.73 (1077.36 to 4214.01) | 5.08 (4.31 to 5.85) |
| Neuroblastoma recurrent | 16 | 767.21 (430.98 to 1365.77) | 763.96 (429.91 to 1357.56) | 4.96 (4.17 to 5.76) |
| Blood pressure diastolic abnormal | 35 | 39.46 (28.20 to 55.22) | 39.10 (28.03 to 54.55) | 4.65 (4.12 to 5.18) |
| Tonic convulsion | 19 | 103.78 (65.37 to 164.75) | 103.26 (65.19 to 163.57) | 4.81 (4.09 to 5.54) |
| Metal poisoning | 15 | 260.35 (151.91 to 446.2) | 259.32 (151.59 to 443.59) | 4.78 (3.96 to 5.60) |
| Urine leukocyte esterase positive | 15 | 68.95 (41.17 to 115.47) | 68.68 (41.09 to 114.79) | 4.42 (3.60 to 5.24) |
| Behaviour disorder | 34 | 22.41 (15.96 to 31.47) | 22.22 (15.87 to 31.10) | 4.08 (3.54 to 4.61) |
| Sinus headache | 21 | 31.20 (20.25 to 48.07) | 31.03 (20.19 to 47.70) | 4.18 (3.49 to 4.87) |
| Blood pressure systolic abnormal | 22 | 28.21 (18.50 to 43.01) | 28.05 (18.44 to 42.67) | 4.12 (3.45 to 4.79) |
| Blood pressure systolic increased | 68 | 15.31 (12.04 to 19.48) | 15.05 (11.88 to 19.07) | 3.76 (3.38 to 4.14) |
| Post viral fatigue syndrome | 11 | 168.88 (91.20 to 312.72) | 168.39 (91.09 to 311.29) | 4.33 (3.36 to 5.30) |

Abbreviations: CBD: cannabidiol; CI, confidence interval; CrI, credible interval; FAERS: U.S. FDA Adverse Event Reporting System; IC, information component; IC_025_: lower bound of the 95% credible interval for IC; PRR, proportional reporting ratio; PT, preferred term; ROR, reporting odds ratio

**Online Appendix 3A.** The top 30 disproportionality analysis estimates for Epidiolex (top panel) and CBD (bottom panel) with seizure indication at the preferred term level, ranked by IC_025_ (FAERS Q2 2018 to Q1 2023)

|  | **Epidiolex IC (95% CrI)** | **CBD IC (95% CrI)** |
| --- | --- | --- |
| **Top 30 PTs by Epidiolex disproportionality** |  |  |
| Seizure cluster | 6.31 (5.98 to 6.65) | NSD |
| Change in seizure presentation | 6.08 (5.69 to 6.48) | 2.75 (0.76 to 4.74) |
| Weight abnormal | 5.77 (5.5 to 6.04) | NSD |
| Atonic seizures | 5.88 (5.46 to 6.3) | NSD |
| Seizure | 5.46 (5.42 to 5.51) | 4.71 (4.47 to 4.95) |
| Emergency care | 5.53 (5.26 to 5.8) | NSD |
| Product supply issue | 5.4 (5.22 to 5.58) | NSD |
| Anticonvulsant drug level increased | 5.37 (4.9 to 5.85) | NSD |
| Product administration interrupted | 4.42 (4.14 to 4.7) | NSD |
| Drooling | 4.25 (3.89 to 4.6) | NSD |
| Sudden unexplained death in epilepsy | 4.59 (3.84 to 5.33) | 3.42 (1.93 to 4.9) |
| Prescribed overdose | 4.01 (3.78 to 4.24) | NSD |
| Product distribution issue | 3.98 (3.63 to 4.34) | NSD |
| Generalised tonic-clonic seizure | 3.76 (3.51 to 4.01) | 4.54 (3.92 to 5.16) |
| Aggression | 3.66 (3.47 to 3.86) | 3.23 (2.41 to 4.05) |
| Therapy responder | 4.26 (3.29 to 5.22) | NSD |
| Status epilepticus | 3.62 (3.27 to 3.97) | 5.17 (4.6 to 5.74) |
| Petit mal epilepsy | 3.57 (3.03 to 4.1) | 4.4 (3.51 to 5.29) |
| Tonic convulsion | 3.81 (2.99 to 4.63) | 5.19 (4.46 to 5.91) |
| Product administered to patient of inappropriate age | 3.37 (2.96 to 3.77) | NSD |
| Head banging | 3.91 (2.94 to 4.88) | NSD |
| Drug withdrawal convulsions | 3.66 (2.91 to 4.41) | 2.65 (0.66 to 4.65) |
| Product use in unapproved indication | 2.96 (2.88 to 3.03) | 1.38 (0.82 to 1.93) |
| Inappropriate affect | 3.69 (2.87 to 4.52) | NSD |
| Screaming | 3.41 (2.84 to 3.98) | 2.82 (1.13 to 4.51) |
| Abnormal behaviour | 3.1 (2.82 to 3.38) | 3.24 (2.31 to 4.16) |
| Myoclonic epilepsy | 3.63 (2.81 to 4.45) | 3.97 (2.82 to 5.12) |
| Psychomotor hyperactivity | 3.16 (2.8 to 3.53) | NSD |
| Lethargy | 3 (2.79 to 3.21) | 3.28 (2.55 to 4) |
| Somnolence | 2.88 (2.78 to 2.99) | 3.53 (3.19 to 3.87) |
| **Top 30 PTs by CBD disproportionality** |  |  |
| Multiple-drug resistance | NSD | 6.67 (6.28 to 7.06) |
| Drug resistance | NSD | 5.09 (4.68 to 5.5) |
| Status epilepticus | 3.62 (3.27 to 3.97) | 5.17 (4.6 to 5.74) |
| Seizure | 5.46 (5.42 to 5.51) | 4.71 (4.47 to 4.95) |
| Tonic convulsion | 3.81 (2.99 to 4.63) | 5.19 (4.46 to 5.91) |
| Epilepsy | 1.65 (1.21 to 2.09) | 4.8 (4.3 to 5.3) |
| Generalised tonic-clonic seizure | 3.76 (3.51 to 4.01) | 4.54 (3.92 to 5.16) |
| Drug interaction | 0.87 (0.62 to 1.12) | 4.21 (3.92 to 4.51) |
| Drug level increased | 1.23 (0.58 to 1.87) | 4.38 (3.71 to 5.06) |
| Petit mal epilepsy | 3.57 (3.03 to 4.1) | 4.4 (3.51 to 5.29) |
| Somnolence | 2.88 (2.78 to 2.99) | 3.53 (3.19 to 3.87) |
| Ataxia | 1.73 (1.03 to 2.44) | 4.03 (3.14 to 4.91) |
| Irritability | 2.69 (2.44 to 2.93) | 3.56 (2.88 to 4.23) |
| Myoclonic epilepsy | 3.63 (2.81 to 4.45) | 3.97 (2.82 to 5.12) |
| Lethargy | 3 (2.79 to 3.21) | 3.28 (2.55 to 4) |
| Aggression | 3.66 (3.47 to 3.86) | 3.23 (2.41 to 4.05) |
| Abnormal behaviour | 3.1 (2.82 to 3.38) | 3.24 (2.31 to 4.16) |
| Therapeutic product ineffective for unapproved indication | NSD | 3.65 (2.3 to 4.99) |
| Therapeutic product effective for unapproved indication | NSD | 3.65 (2.3 to 4.99) |
| Hepatitis fulminant | NSD | 3.49 (2.14 to 4.83) |
| Sudden unexplained death in epilepsy | 4.59 (3.84 to 5.33) | 3.42 (1.93 to 4.9) |
| Infantile spasms | 2.11 (0.12 to 4.11) | 3.41 (1.92 to 4.9) |
| Drug ineffective for unapproved indication | 0.62 (0.2 to 1.05) | 2.62 (1.8 to 3.44) |
| Hyperammonaemic encephalopathy | NSD | 3.29 (1.8 to 4.78) |
| Decreased appetite | 1.71 (1.56 to 1.86) | 2.23 (1.74 to 2.72) |
| Hypersomnia | 2.95 (2.66 to 3.23) | 2.8 (1.72 to 3.87) |
| Alanine aminotransferase increased | NSD | 2.6 (1.68 to 3.52) |
| Treatment failure | NSD | 2.26 (1.59 to 2.93) |
| Acute respiratory failure | 0.86 (0.17 to 1.55) | 2.7 (1.46 to 3.93) |
| Sedation | 2.94 (2.63 to 3.26) | 2.64 (1.4 to 3.87) |

Abbreviations: CrI*,* credible interval; FAERS: U.S. FDA Adverse Event Reporting System; IC: information component; PT*,* preferred term; NSD, no signal detected (lower bound of the 95% credible interval for the information component IC_025_ ≤0). Seizure-related indication was classified at the case level using an approximation of the MedDRA Standardised MedDRA Query (SMQ) "Convulsions" (narrow and broad scope), matching reported indications containing the following terms: seizure, convulsion, epilep*, Lennox-Gastaut, Dravet, tuberous sclerosis, status epilepticus, or infantile spasms.

**Online Appendix 3B.** The top 30 disproportionality analysis estimates for Epidiolex (top panel) and CBD (bottom panel) without seizure indication, at the preferred term level, ranked by IC_025_ (FAERS Q2 2018 to Q1 2023)

|  | **Epidiolex IC (95% CrI)** | **CBD IC (95% CrI)** |
| --- | --- | --- |
| **Top 30 PTs by Epidiolex disproportionality** |  |  |
| Product administration interrupted | 5.96 (5.61 to 6.3) | 2.01 (0.78 to 3.25) |
| Seizure | 5.45 (5.35 to 5.56) | 1.47 (1.06 to 1.87) |
| Product supply issue | 3.99 (3.33 to 4.65) | NSD |
| Brain operation | 4.04 (3.08 to 5.01) | NSD |
| Atonic seizures | 4.09 (3.01 to 5.17) | NSD |
| Generalised tonic-clonic seizure | 3.58 (2.98 to 4.19) | NSD |
| Anticonvulsant drug level increased | 4.05 (2.97 to 5.13) | NSD |
| Seizure cluster | 4.04 (2.96 to 5.12) | NSD |
| Aggression | 3.03 (2.45 to 3.6) | 1.53 (0.74 to 2.32) |
| Product administered to patient of inappropriate age | 3.32 (2.43 to 4.2) | NSD |
| Sedation | 3.12 (2.43 to 3.8) | NSD |
| Prescribed overdose | 3.06 (2.32 to 3.81) | NSD |
| Behaviour disorder | 3.16 (2.19 to 4.13) | 4.11 (3.54 to 4.68) |
| Therapy change | 3.08 (2.15 to 4) | NSD |
| Hospitalisation | 2.43 (2.09 to 2.76) | NSD |
| Drooling | 2.99 (1.84 to 4.14) | NSD |
| Weight abnormal | 3.06 (1.72 to 4.41) | NSD |
| Product distribution issue | 2.82 (1.67 to 3.97) | NSD |
| Incorrect route of product administration | 2.4 (1.61 to 3.2) | NSD |
| Liquid product physical issue | 2.7 (1.47 to 3.94) | NSD |
| Status epilepticus | 2.53 (1.38 to 3.68) | NSD |
| Product dose omission issue | 1.66 (1.38 to 1.95) | NSD |
| Somnolence | 1.74 (1.33 to 2.15) | 1.72 (1.39 to 2.05) |
| Aspiration | 2.55 (1.31 to 3.79) | NSD |
| Diarrhoea | 1.47 (1.24 to 1.69) | 1.65 (1.48 to 1.82) |
| Reaction to excipient | 2.66 (1.17 to 4.15) | NSD |
| Anger | 2.09 (1.17 to 3.01) | 1.99 (1.22 to 2.76) |
| Lethargy | 1.88 (1.12 to 2.65) | 2.15 (1.6 to 2.71) |
| Aura | 2.8 (1.11 to 4.49) | 2.25 (0.26 to 4.24) |
| Abnormal behaviour | 2.03 (1.06 to 3) | 1.88 (1.06 to 2.7) |
| **Top 30 PTs by CBD disproportionality** |  |  |
| Blood pressure diastolic decreased | NSD | 5.1 (4.71 to 5.49) |
| Device related thrombosis | NSD | 5.1 (4.46 to 5.74) |
| Malignant cranial nerve neoplasm | NSD | 5.1 (4.34 to 5.87) |
| Retro-orbital neoplasm | NSD | 5.09 (4.32 to 5.86) |
| Blood pressure diastolic abnormal | NSD | 4.83 (4.3 to 5.36) |
| Neuroblastoma recurrent | NSD | 4.98 (4.18 to 5.77) |
| Metal poisoning | NSD | 4.81 (3.99 to 5.63) |
| Urine leukocyte esterase positive | NSD | 4.5 (3.68 to 5.32) |
| Sinus headache | NSD | 4.34 (3.65 to 5.03) |
| Blood pressure systolic increased | NSD | 4.02 (3.64 to 4.4) |
| Blood pressure systolic abnormal | NSD | 4.29 (3.62 to 4.96) |
| Behaviour disorder | 3.16 (2.19 to 4.13) | 4.11 (3.54 to 4.68) |
| Large intestine polyp | NSD | 4.09 (3.51 to 4.66) |
| Finger deformity | NSD | 4.16 (3.48 to 4.85) |
| Metastases to spine | NSD | 4.19 (3.42 to 4.96) |
| Post viral fatigue syndrome | NSD | 4.37 (3.4 to 5.33) |
| Infusion site pruritus | NSD | 4.13 (3.36 to 4.9) |
| Nail disorder | NSD | 3.94 (3.3 to 4.58) |
| Metastases to liver | NSD | 3.74 (3.25 to 4.23) |
| Body temperature decreased | NSD | 3.77 (3.22 to 4.31) |
| Infusion site scar | NSD | 4.21 (3.19 to 5.22) |
| Psychomotor hyperactivity | NSD | 3.73 (3.18 to 4.28) |
| Product formulation issue | NSD | 3.85 (3.13 to 4.58) |
| Culture urine positive | NSD | 4.1 (3.08 to 5.12) |
| Urine abnormality | NSD | 3.81 (3.04 to 4.58) |
| Neutrophil count increased | NSD | 3.67 (3.02 to 4.33) |
| Microsporidia infection | NSD | 4.08 (3 to 5.16) |
| Nerve compression | NSD | 3.6 (2.93 to 4.27) |
| Pyelonephritis chronic | NSD | 3.99 (2.84 to 5.14) |
| Heart rate irregular | NSD | 3.33 (2.81 to 3.85) |

Abbreviations: CrI*,* credible interval; FAERS: U.S. FDA Adverse Event Reporting System; IC: information component; PT*,* preferred term; NSD, no signal detected (lower bound of the 95% credible interval for the information component IC_025_ ≤0). Seizure-related indication was classified at the case level using an approximation of the MedDRA Standardised MedDRA Query (SMQ) "Convulsions" (narrow and broad scope), matching reported indications containing the following terms: seizure, convulsion, epilep*, Lennox-Gastaut, Dravet, tuberous sclerosis, status epilepticus, or infantile spasms.

**Online Appendix 4. R code for CDP Terminology Identification in FAERS**

#################################################################################

#

# Title: A Feasibility Assessment of the FDA Adverse Event Reporting System for

# the Detection of Cannabis-Related Safety Signals

#

# Authors: Priscilla O. M. V. Lopes, Cory S. Harris, Christopher A. Gravel

#

# This script identifies and extracts cannabis-derived product (CDP)

# terminology from the FAERS drug name field using a predefined set of

# character strings associated with pharmaceutical and non-pharmaceutical

# cannabinoid formulations. Due to the lack of standardized nomenclature

# for CDPs in FAERS, this approach searches for partial string matches

# to broadly capture cannabinoid-related terms, including full chemical

# names, brand names, misspellings, abbreviations, and informal or

# international variants.

#

# Approach:

# Step 1 - Data setup: Download FAERS/LAERS quarterly data from:

# https://fis.fda.gov/extensions/FPD-QDE-FAERS/FPD-QDE-FAERS.html

# Extract the DRUG file (e.g., DRUG25Q1.txt), load it into R as

# a data.table, and set the column to search. The DRUG file

# contains two drug name columns: 'drugname' and 'prod_ai'.

# This study used 'drugname' (set via drug_col below).

#

# Step 2 - String search: 14 predefined character strings are searched

# against the specified column (case-insensitive). These

# include partial matches for chemical names (e.g., 'cannab'

# captures cannabidiol, tetrahydrocannabinol, cannabis), brand

# names (e.g., 'epidiol' for Epidiolex, 'sative' for Sativex),

# and common abbreviations (e.g., 'thc', 'cbd').

#

# Step 3 - Manual review: each matched term is reviewed for sensitivity

# and specificity. Terms are either:

# (a) excluded if unrelated (e.g., non-cannabinoid botanicals)

# or ambiguous, or

# (b) mapped to a CDP exposure group based on active ingredient

# and product quality control standards.

#

# Step 4 - Exposure group assignment: terms are classified into one of

# seven predefined groups:

# - Epidiolex: branded cannabidiol (pharmaceutical)

# - Sativex: nabiximols (pharmaceutical)

# - Rx THC: dronabinol, nabilone (pharmaceutical)

# - CBD: non-pharmaceutical cannabidiol

# - THC: non-pharmaceutical delta-8/delta-9-THC

# - THC/CBD: reports mentioning both THC and CBD (non-pharmaceutical)

# - Cannabis: general terms (cannabis, marijuana, cannabinoids) (non-pharmaceutical)

#

# Output:

# - CDP_preliminary_mapping_for_manual_review.csv: a two-column file

# (term, group) listing each matched term and its preliminary

# exposure group assignment.

##################################################################################

# Install packages

library(data.table)

# Load the FAERS/LAERS drug file as a data.table

# Example: drug_dt <- fread("DRUG25Q1.txt", sep = "$", quote = "")

# Set the drug name column to search (e.g., "drugname")

drug_col <- "drugname"

# Define search terms for cannabinoid-derived products

cdp_terms <- c("cannab", "canab", "mariju", "marih",

"thc", "cbd", "nabixi", "dronab",

"nabilo", "cesam", "syndros", "marino",

"epidiol", "sative")

# Search for matches in the specified column (case-insensitive)

cdp_matches <- c()

for (i in 1:length(cdp_terms)) {

temp <- unique(drug_dt[grepl(toupper(cdp_terms[i]), toupper(get(drug_col))), get(drug_col)])

cdp_matches <- c(cdp_matches, temp)

}

# Deduplicate

cdp_matches <- unique(cdp_matches)

# Summary

cat("Number of unique CDP-related drug names identified:",

length(cdp_matches), "\n")

# Preliminary automated mapping to CDP exposure groups

# Create output table

cdp_output <- data.table(term = cdp_matches, group = NA_character_)

# Pharmaceutical CDPs

cdp_output[grepl("epidiol|epidyol", term, ignore.case = TRUE), group := "Epidiolex"]

cdp_output[grepl("sative|nabixi", term, ignore.case = TRUE), group := "Sativex"]

cdp_output[grepl("dronab|nabilo|cesam|syndros|marino", term, ignore.case = TRUE), group := "Rx THC"]

# Non-pharmaceutical CDPs

cdp_output[is.na(group) & grepl("thc|tetrahydrocannabinol", term, ignore.case = TRUE) &

grepl("cbd|cannabidiol", term, ignore.case = TRUE), group := "THC/CBD"]

cdp_output[is.na(group) & grepl("thc", term, ignore.case = TRUE), group := "THC"]

cdp_output[is.na(group) & grepl("cbd|cannabidiol", term, ignore.case = TRUE), group := "CBD"]

# Remaining cannabinoid terms default to Cannabis

cdp_output[is.na(group), group := "Cannabis"]

# Summary of preliminary mapping

cat("\nPreliminary automated mapping:\n")

print(cdp_output[, .N, by = group][order(-N)])

# Export for manual review

fwrite(cdp_output, "CDP_preliminary_mapping_for_manual_review.csv")
